# Supplementary material for: 2-Hydroxyoleic Acid as a Self-Assembly Inducer for Anti-Cancer Drug-Centered Nanoparticles
Source: Pharmaceuticals (Basel). 2023 May 9;16(5):722. doi: 10.3390/ph16050722 (PMC10220756; doi:10.3390/ph16050722)
Supplement: Supplementary file 1 [file pharmaceuticals-16-00722-s001.zip › pharmaceuticals-2327400-supplementary.pdf]

SUPPORTING INFORMATION

2-Hydroxyoleic acid as a self-assembly inducer for anti-cancer drug-centered nanoparticles

Antonia I. Antoniou[1], Giulia Nordio[2], Maria Luisa Di Paolo[3], Eleonora Colombo[1], Beatrice Gaffuri[1], Laura Polito[4], Arianna Amenta[1], Pierfausto Seneci[1], Lisa Dalla Via[2,*] Dario Perdicchia[1,*] and Daniele Passarella[1]

[1] Dipartimento di Chimica, Università degli Studi di Milano, Via Golgi 19, 20133 Milano, Italy; antonia.antoniou@unimi.it (A.I.A.)
[2] Dipartimento di Scienze del Farmaco, Università degli Studi di Padova, Via F. Marzolo 5, 35131 Padova, Italy
[3] Dipartimento di Medicina Molecolare, Università degli studi di Padova, Via G. Colombo 3, 35131 Padova, Italy
[4] Istituto di Scienze e Tecnologie Chimiche "Giulio Natta", SCITEC-CNR, Via G. Fantoli 16/15, 20138 Milano, Italy

* Correspondence: E-mail Addresses: dario.perdicchia@unimi.it; Tel.: +39 02503 14155 (D. Perdicchia), lisa.dallavia@unipd.it; Tel.: +39 04982 75712 (L. Dalla Via)

Table of contents

Figure S1. ^{1}H-NMR spectrum of compound 6. 4

Figure S2. ^{13}C-NMR spectrum of compound 6. 4

Figure S3. ^{1}H-NMR spectrum of compound 14. 5

Figure S4. ^{13}C-NMR spectrum of compound 14. 5

Figure S5. ^{1}H-NMR spectrum of compound 15a. 6

Figure S6. ^{13}C-NMR spectrum of compound 15a. 6

Figure S7. ^{1}H-NMR spectrum of compound 15b. 7

Figure S8. ^{13}C-NMR spectrum of compound 15b. 7

Figure S9. ^{1}H-NMR spectrum of compound 16a. 8

Figure S10. ^{13}C-NMR spectrum of compound 16a. 8

Figure S11. ^{1}H-NMR spectrum of compound 16b. 9

Figure S12. ^{13}C-NMR spectrum of compound 16b. 9

Figure S13. ^{1}H-NMR spectrum of compound 17a. 10

Figure S14. ^{13}C-NMR spectrum of compound 17a. 10

Figure S15. ^{1}H-NMR spectrum of compound 17b. 11

Figure S16. ^{13}C-NMR spectrum of compound 17b. 11

Figure S17. ^{1}H-NMR spectrum of compound 18a. 12

Figure S18. ^{13}C-NMR spectrum of compound 18a. 12

Figure S19. ^{1}H-NMR spectrum of compound 18b. 13

Figure S20. ^{13}C-NMR spectrum of compound 18b. 13

Figure S21. ^{1}H-NMR spectrum of compound 19a. 14

Figure S22. ^{13}C-NMR spectrum of compound 19a. 14

Figure S23. ^{1}H-NMR spectrum of compound 19b. 15

Figure S24. ^{13}C-NMR spectrum of compound 19b. 15

Figure S25. ^{1}H-NMR spectrum of compound 20a. 16

Figure S26. ^{13}C-NMR spectrum of compound 20a. 16

Figure S27. ^{1}H-NMR spectrum of compound 20b. 17

Figure S28. ^{13}C-NMR spectrum of compound 20b. 17

Figure S29. ^{1}H-NMR spectrum of compound 21a. 18

Figure S30. ^{13}C-NMR spectrum of compound 21a. 18

Figure S31. ^{1}H-NMR spectrum of compound 21b. 19

Figure S32. ^{13}C-NMR spectrum of compound 21b. 19

Figure S33. ^{1}H-NMR spectrum of compound 22a. 20

Figure S34. ^{13}C-NMR spectrum of compound 22a. 20

Figure S35. ^{1}H-NMR spectrum of compound 22b. 21

Figure S36. ^{13}C-NMR spectrum of compound 22 21

Figure S37. Cytotoxicity curves of compound 7, 10-13 and the corresponding nanoconjugates on MSTO-211H, HT-29 and LN229. 23

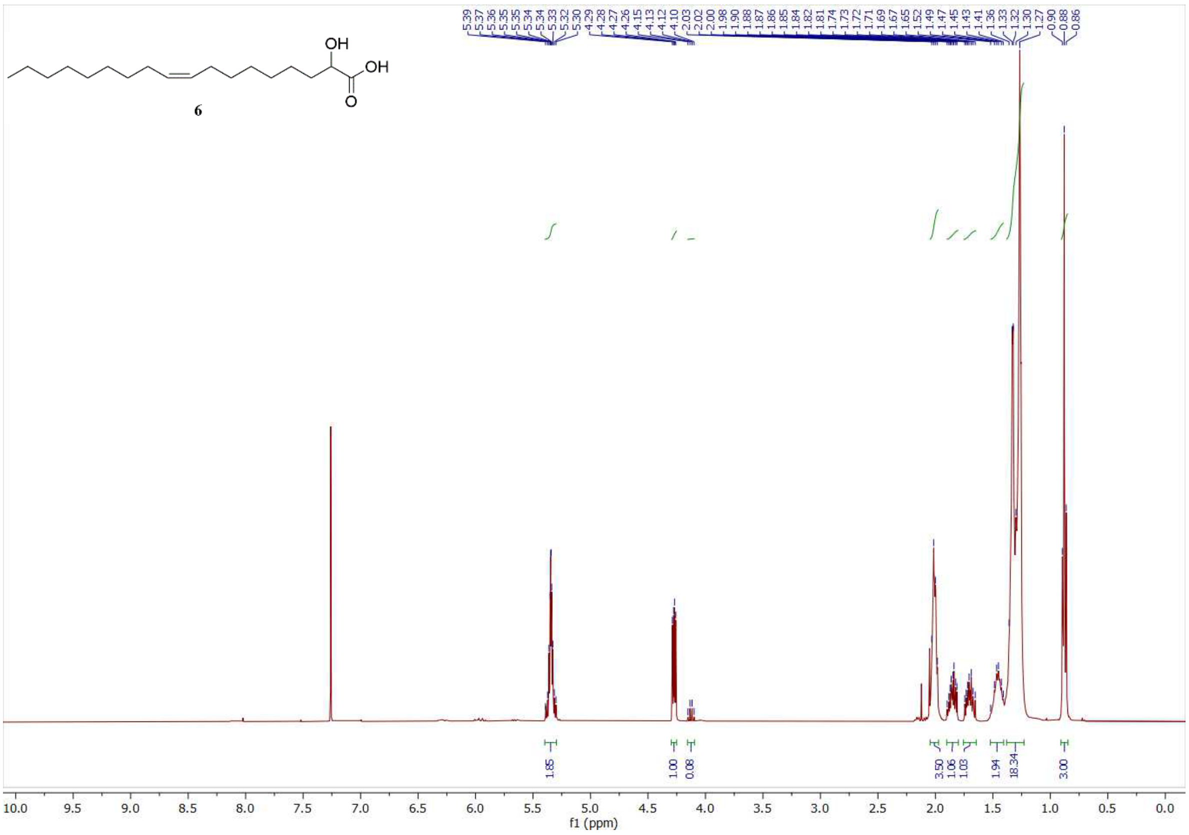

Figure S1. ^{1}H-NMR spectrum of compound 6.

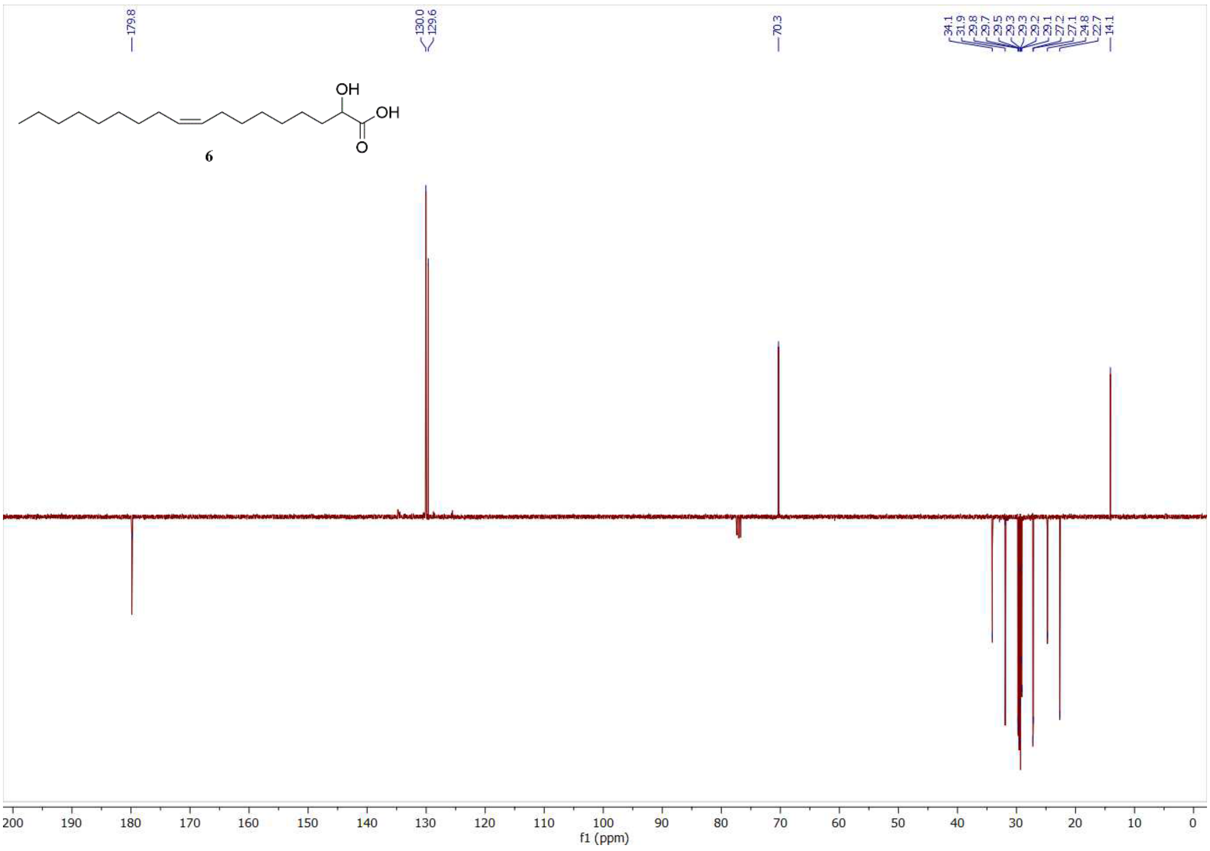

Figure S2. ^{13}C-NMR spectrum of compound 6.

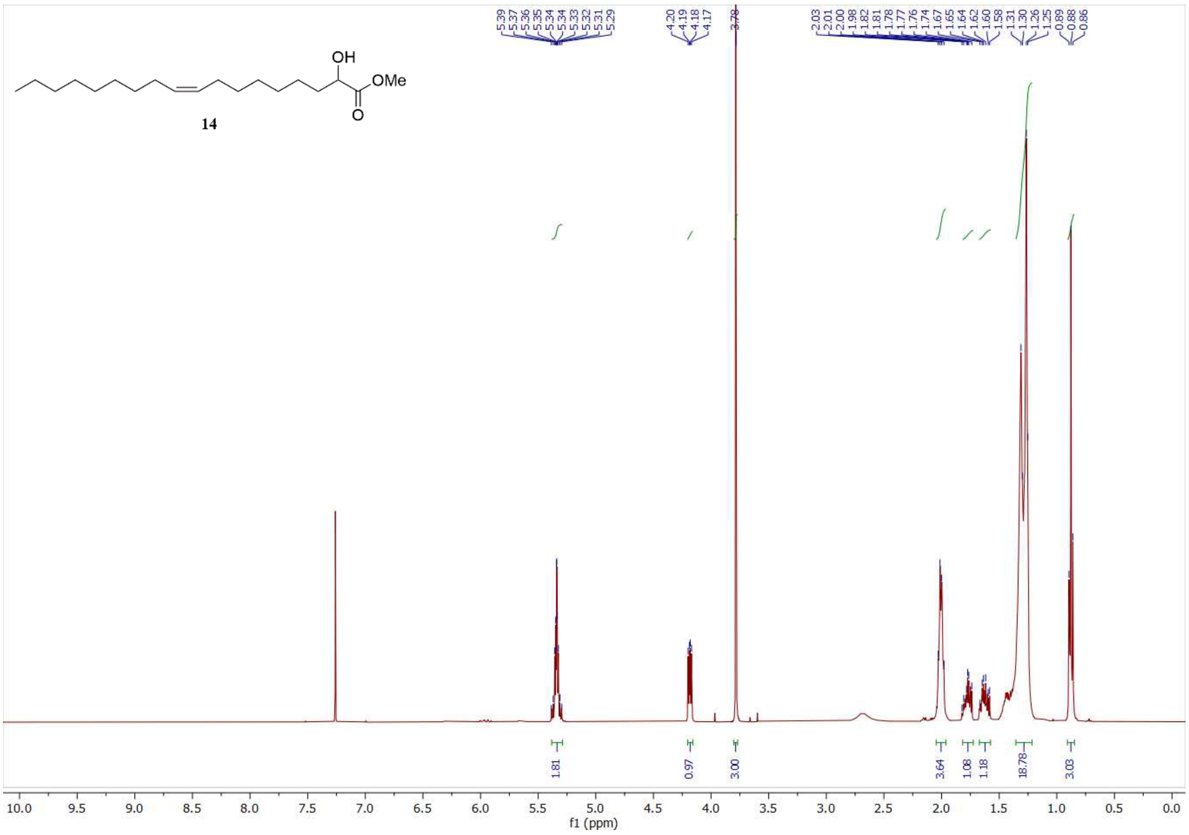

Figure S3. 1H-NMR spectrum of compound 14.

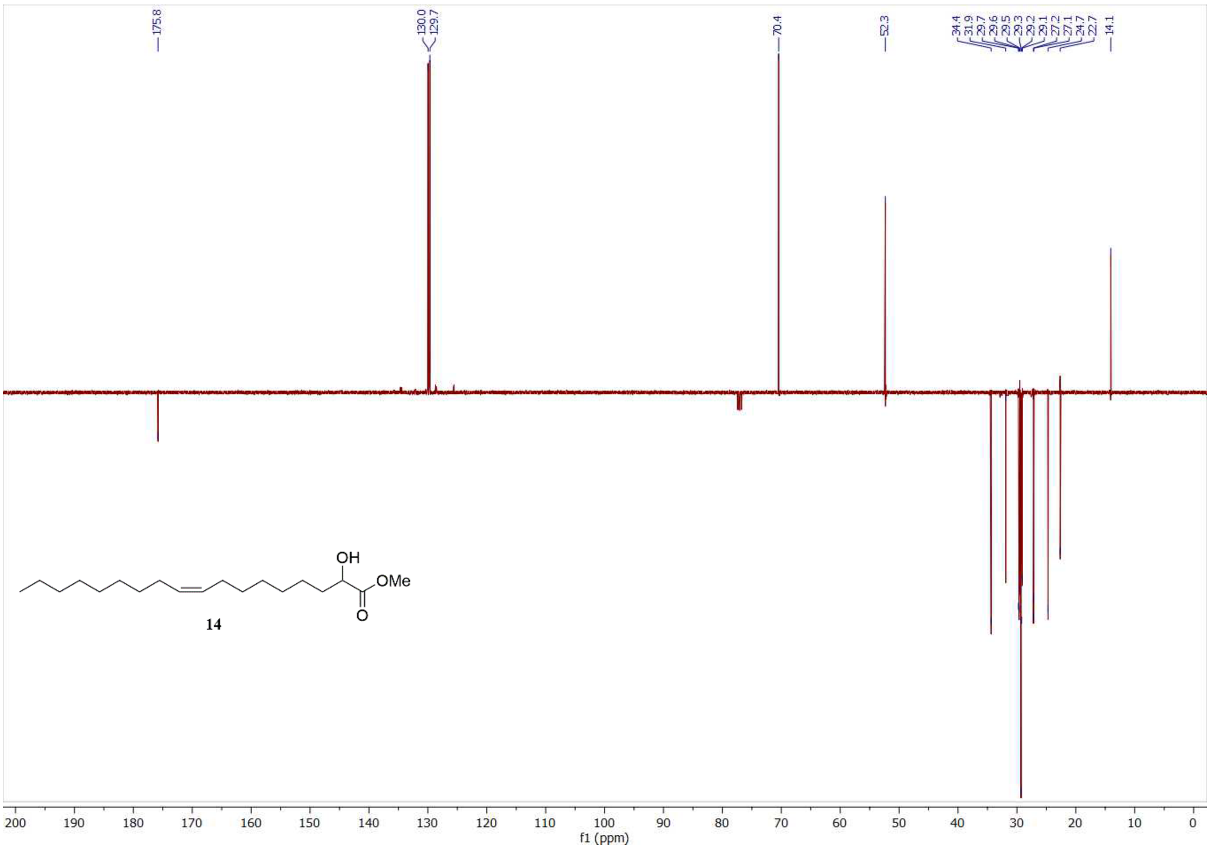

Figure S4. 13C-NMR spectrum of compound 14.

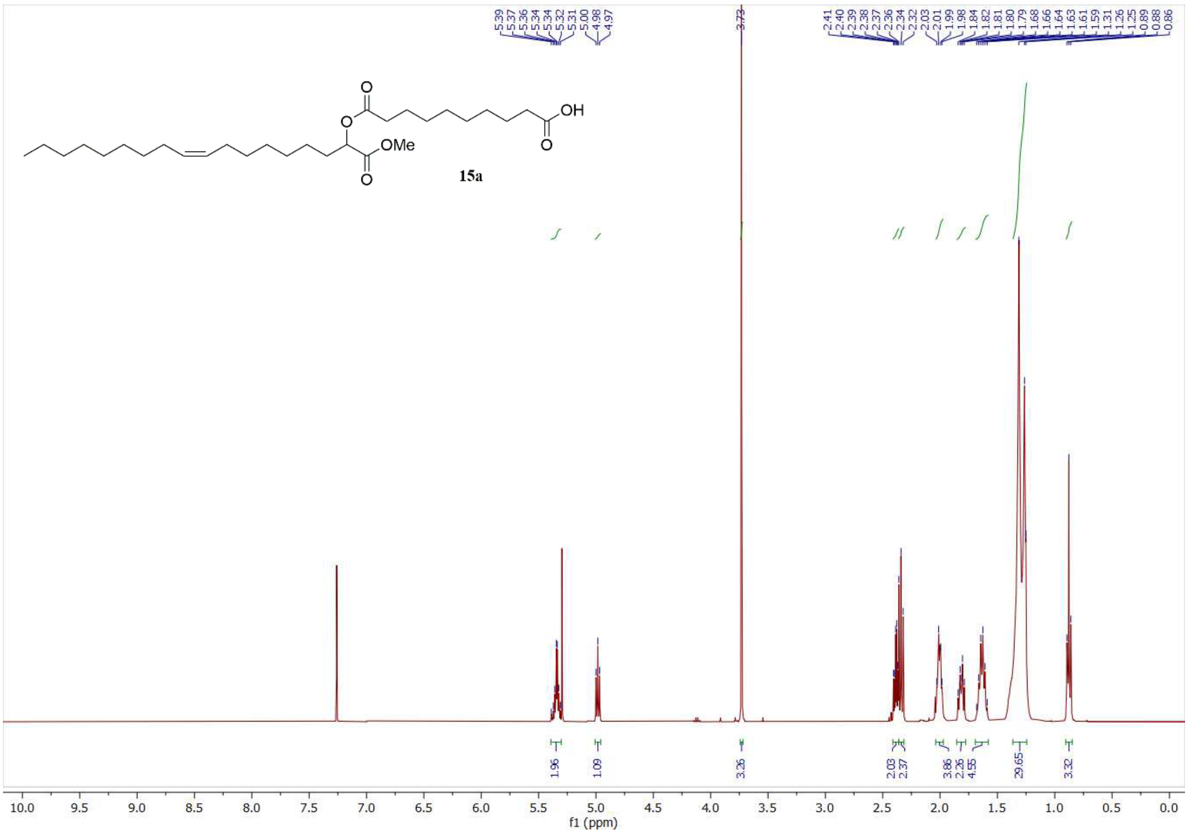

Figure S5. **^{1}H-NMR spectrum of compound 15a.**

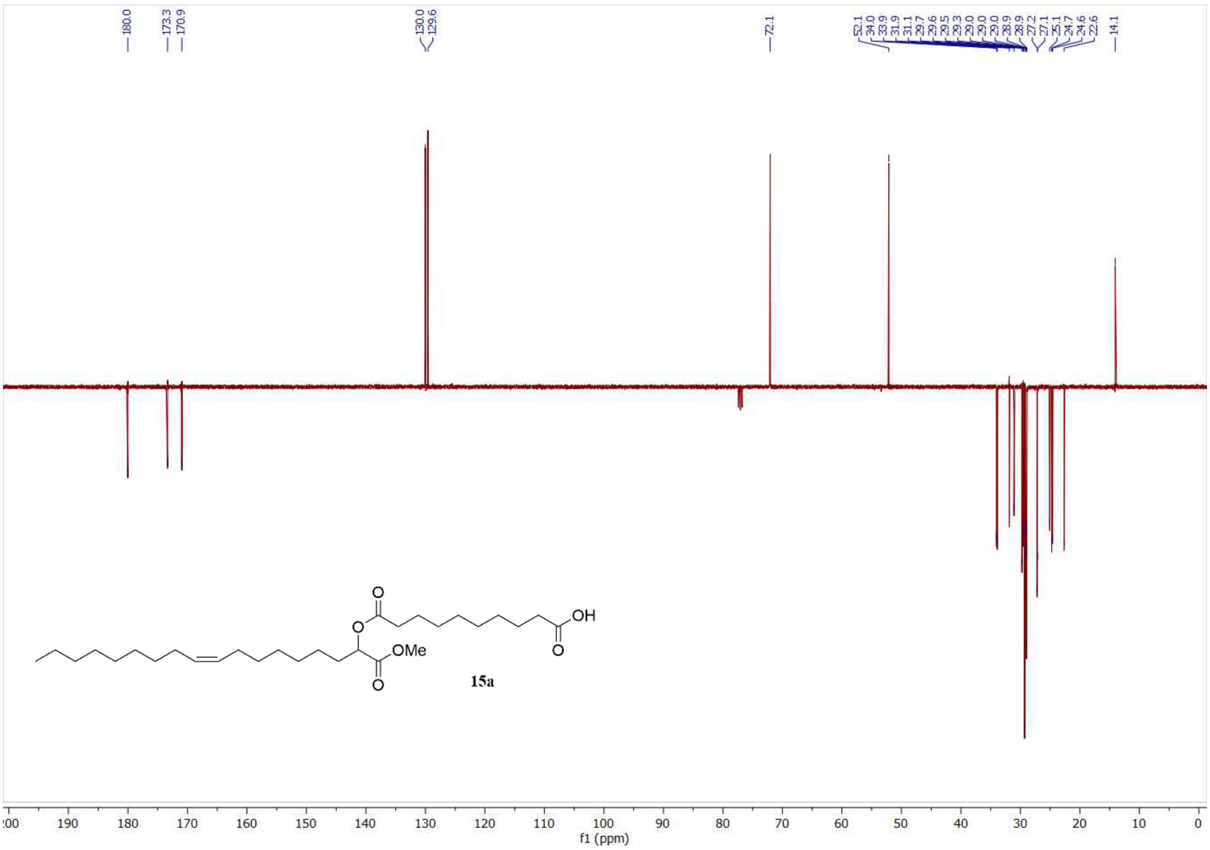

Figure S6. **^{13}C-NMR spectrum of compound 15a.**

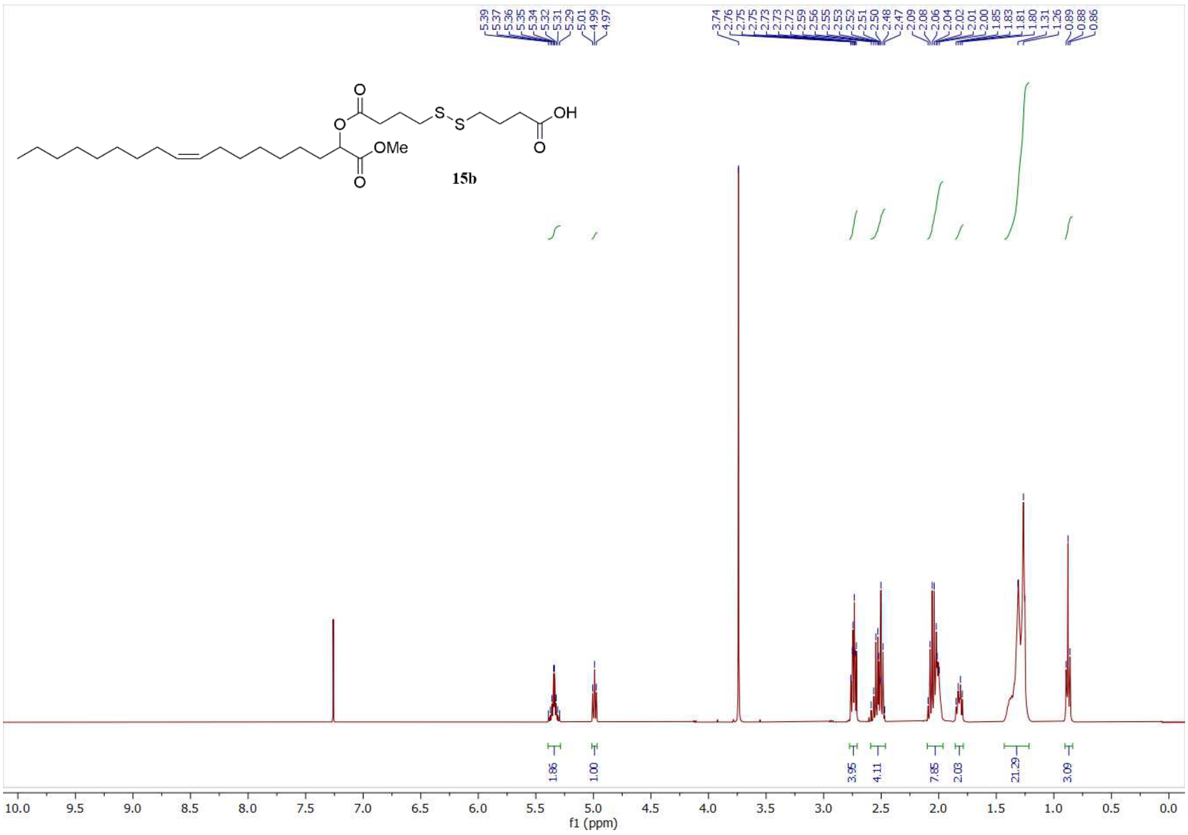

Figure S7. ^{1}H-NMR spectrum of compound 15b.

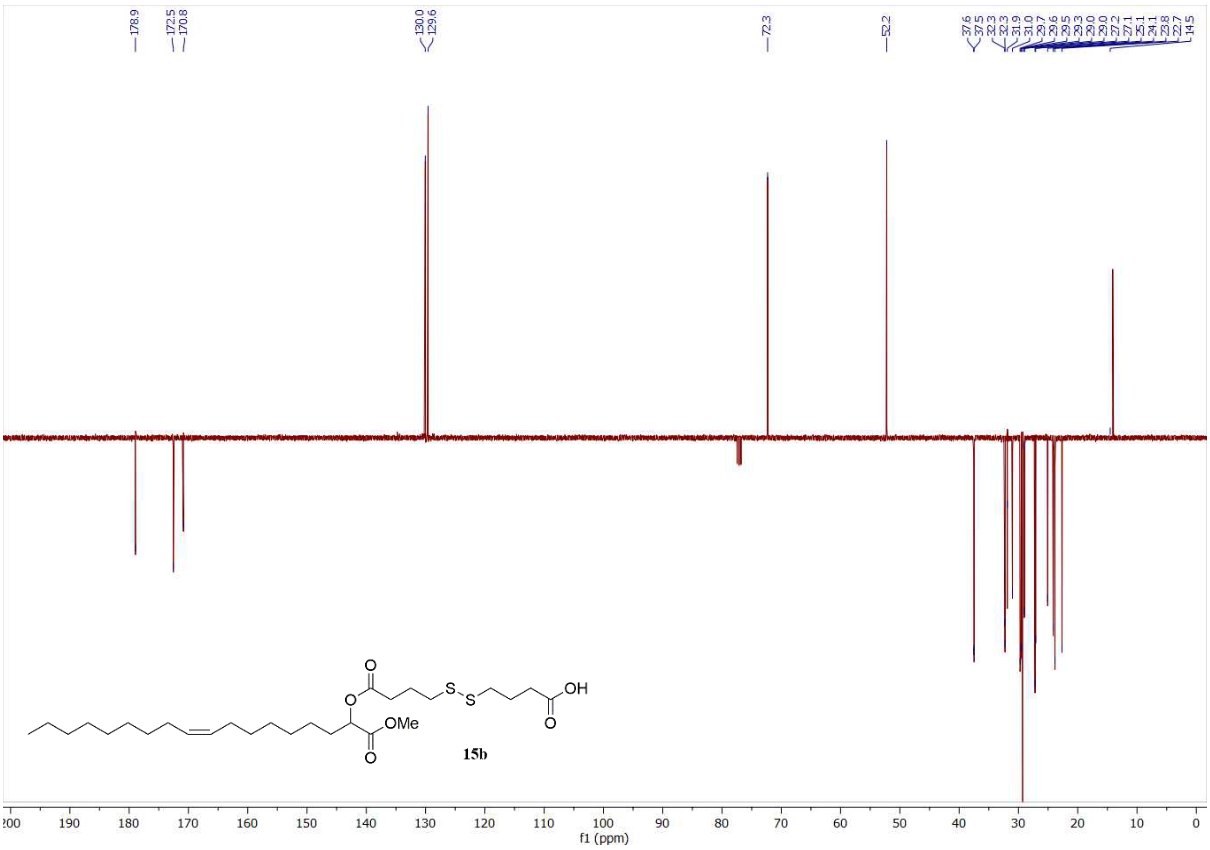

Figure S8. ^{13}C-NMR spectrum of compound 15b.

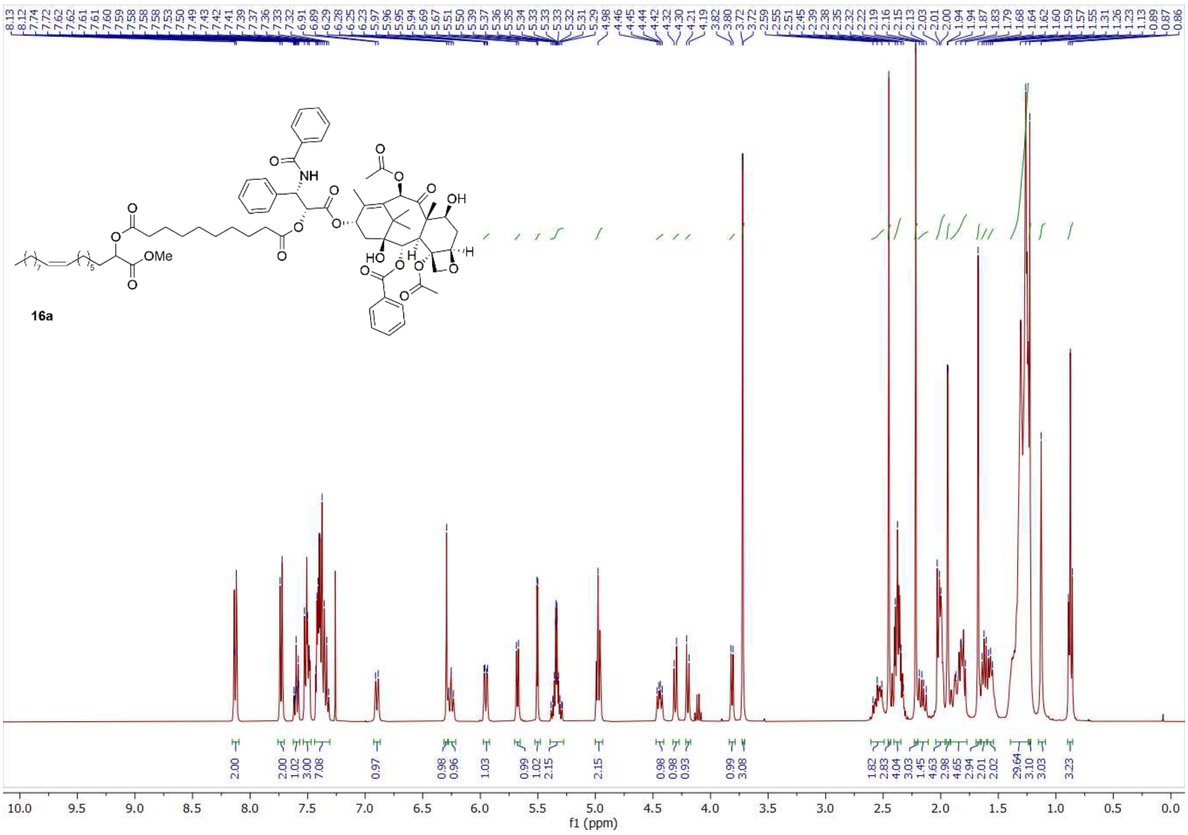

Figure S9. ¹H-NMR spectrum of compound 16a.

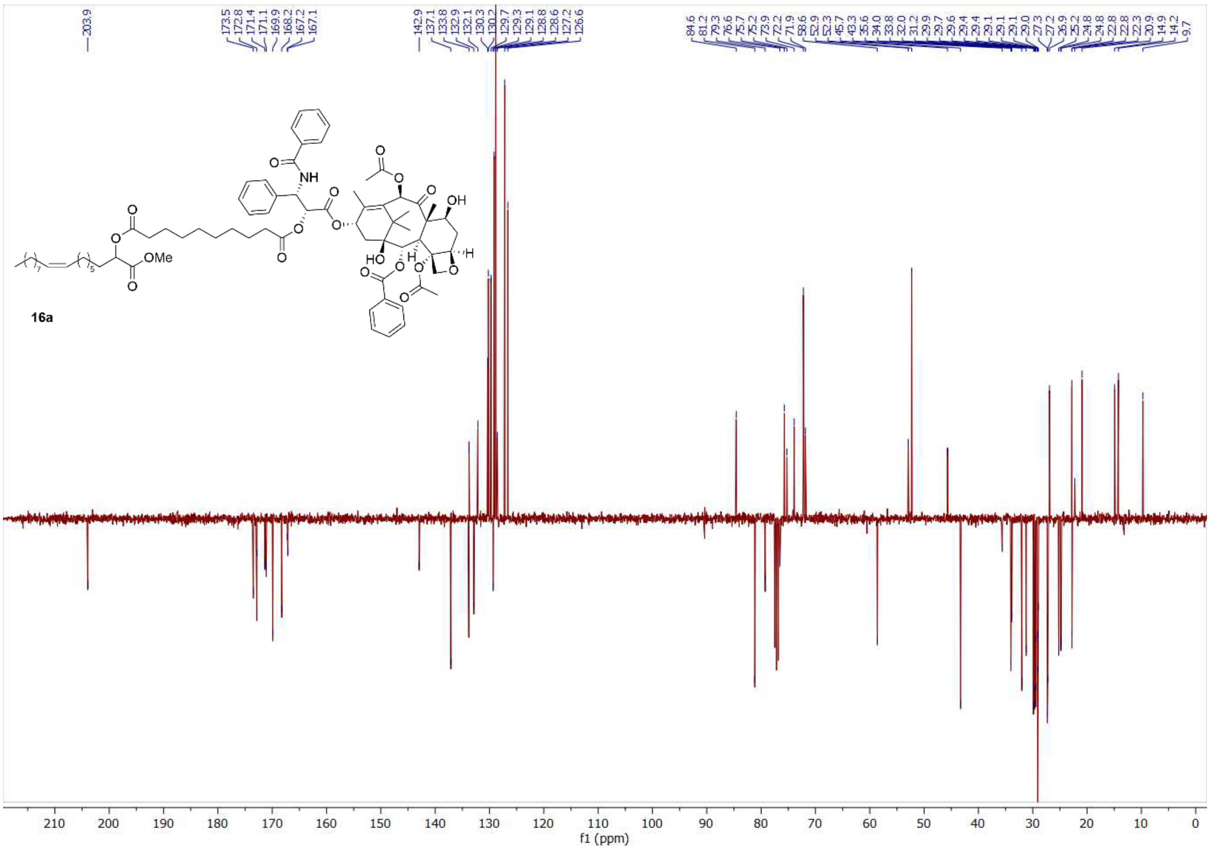

Figure S10. ¹³C-NMR spectrum of compound 16a.

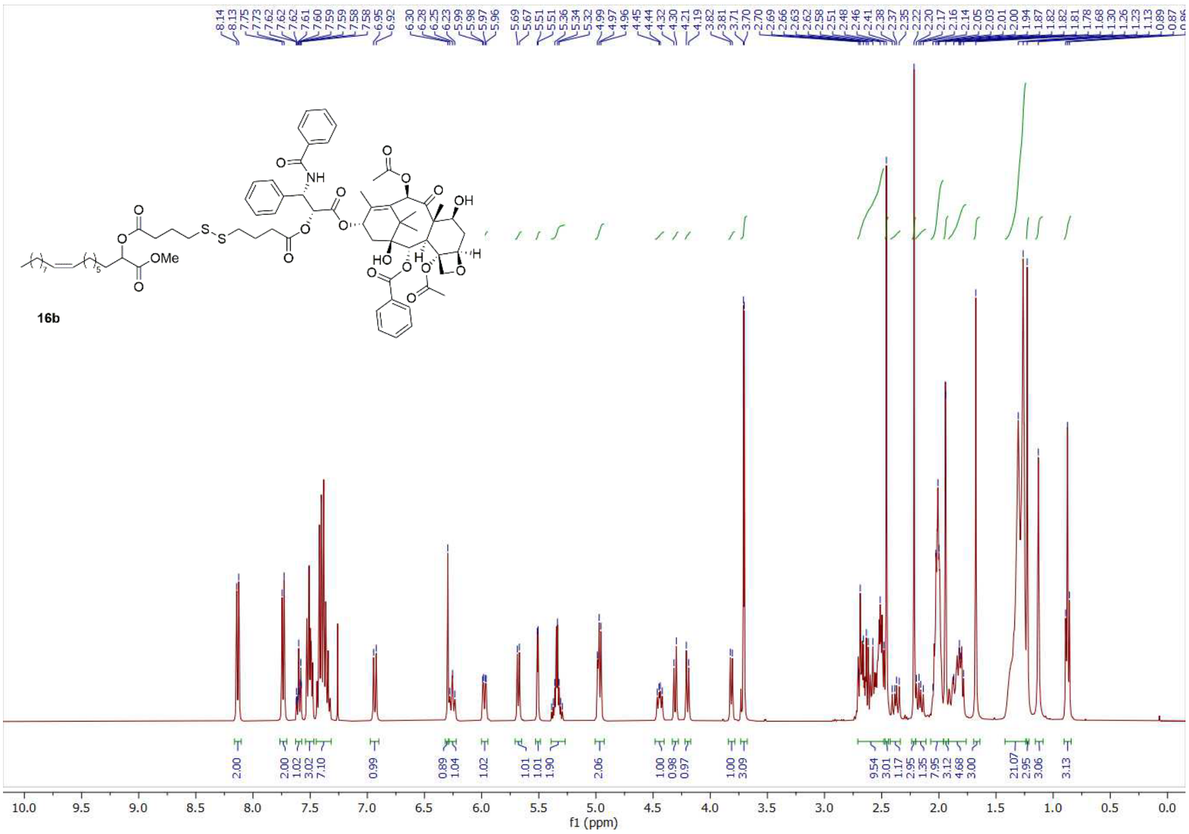

Figure S11. ¹H-NMR spectrum of compound 16b.

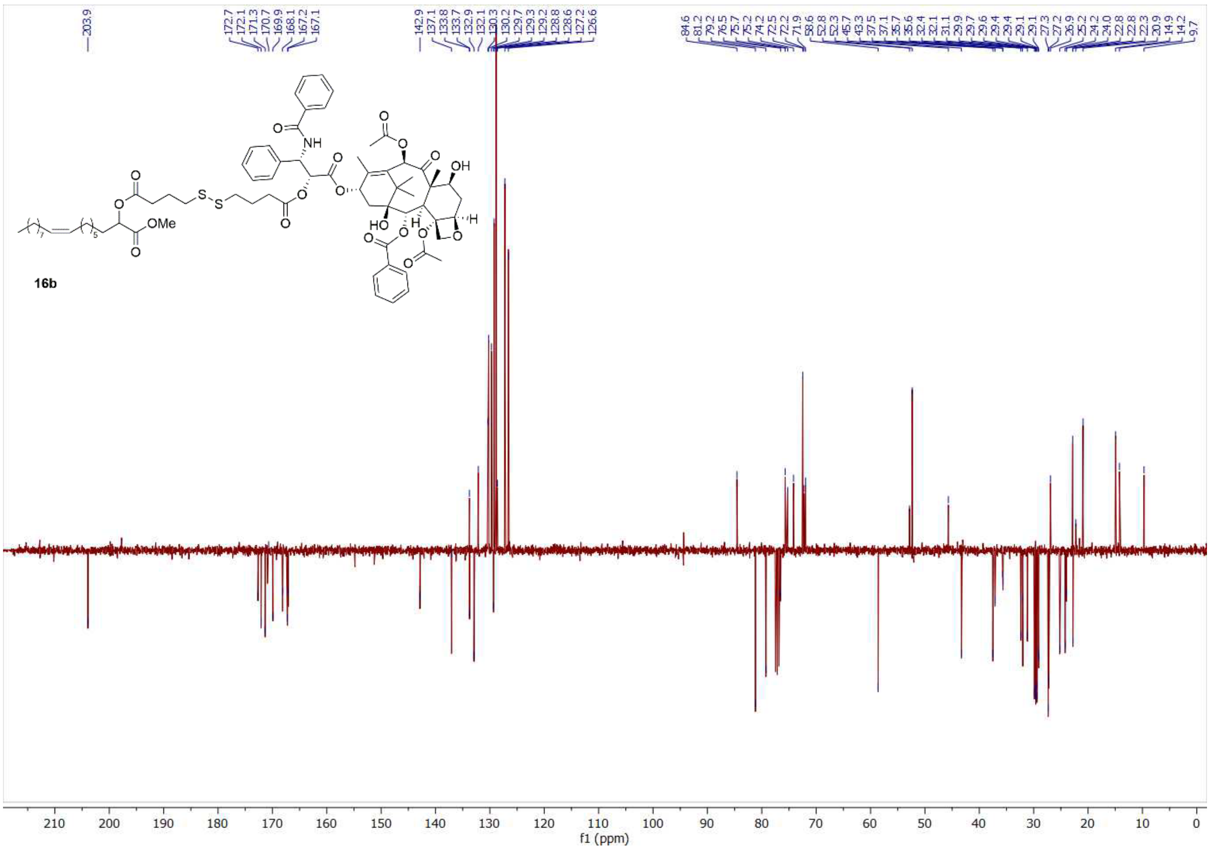

Figure S12. ¹³C-NMR spectrum of compound 16b.

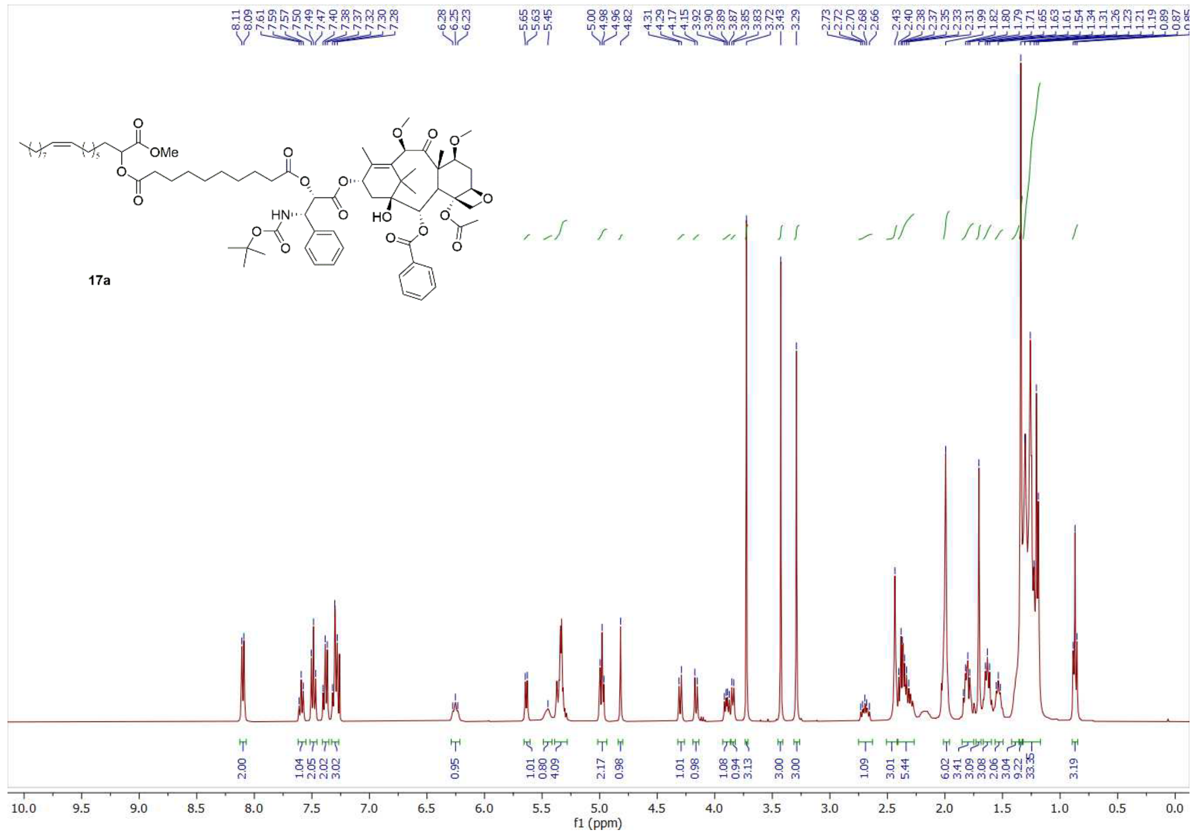

Figure S13. ^{1}H-NMR spectrum of compound 17a.

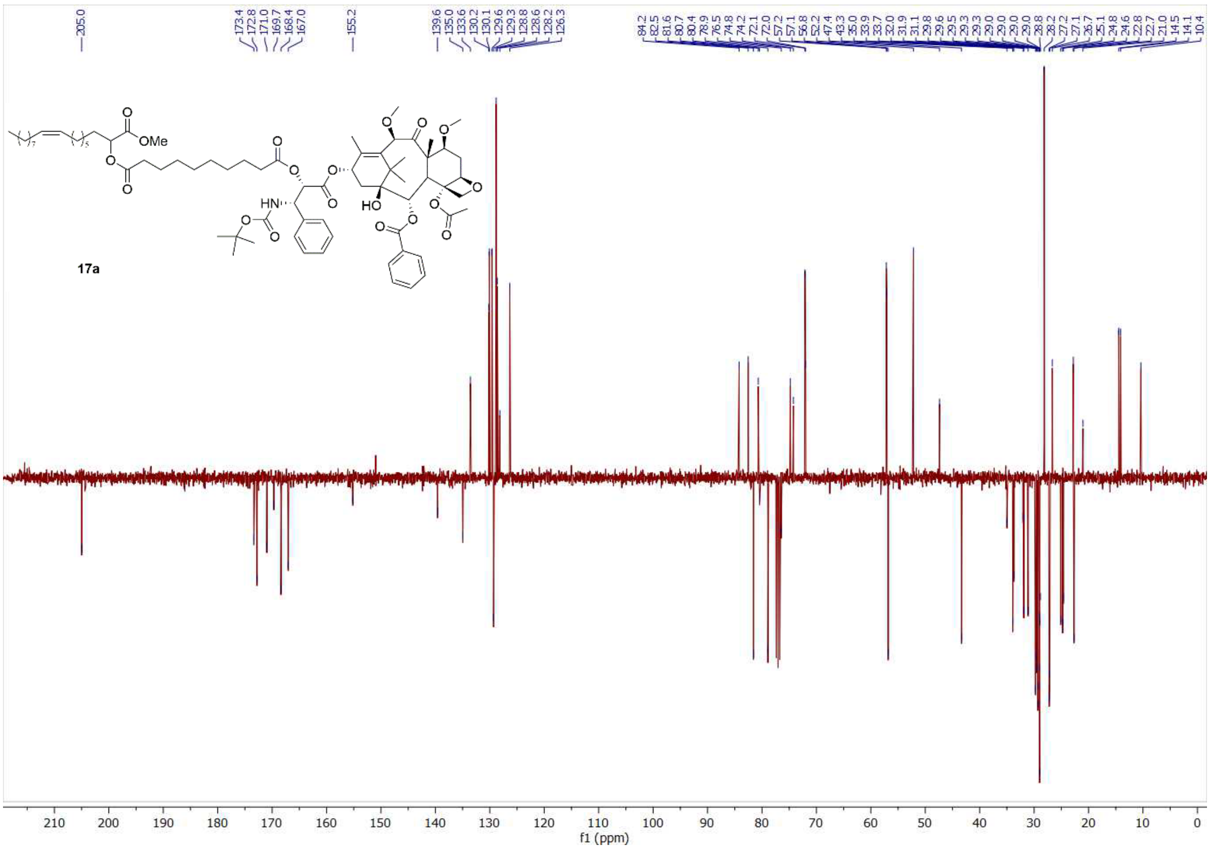

Figure S14. ^{13}C-NMR spectrum of compound 17a.

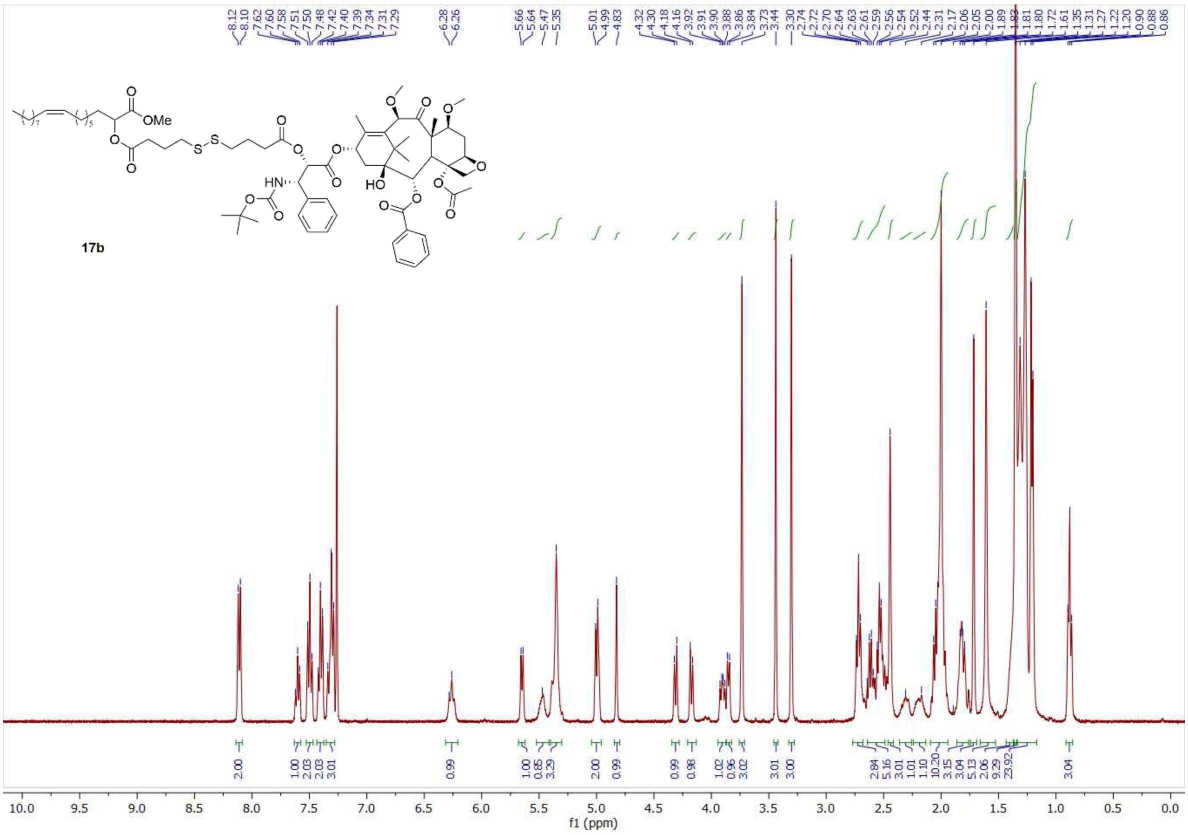

Figure S15. ^{1}**H-NMR spectrum of compound 17b.**

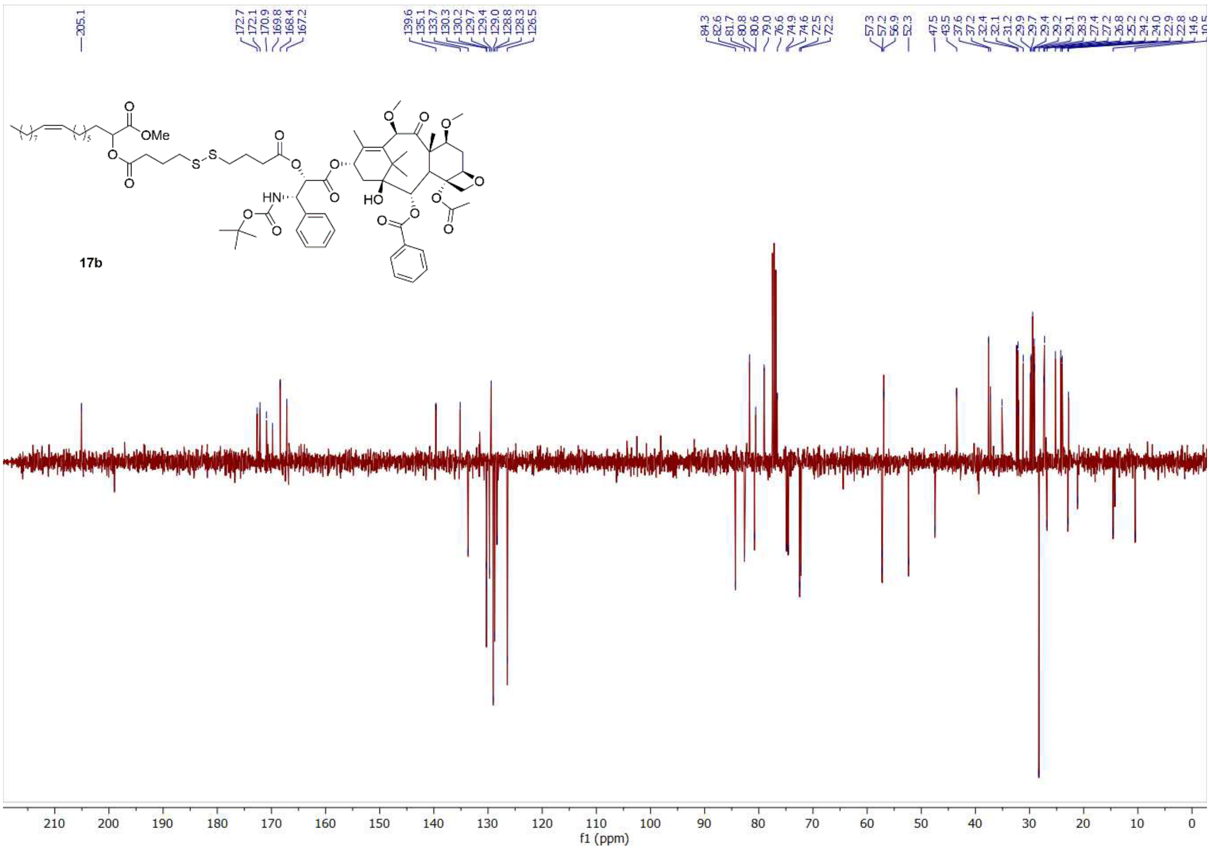

Figure S16. ^{13}**C-NMR spectrum of compound 17b.**

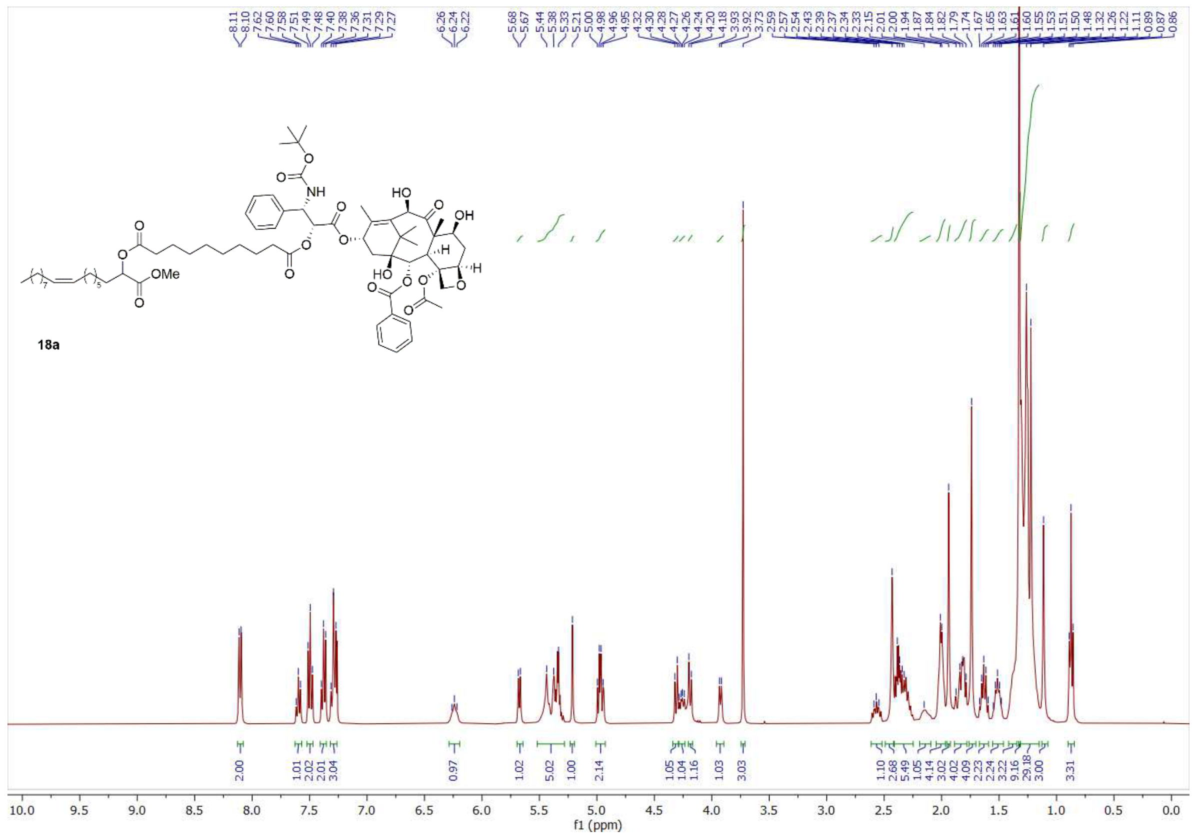

Figure S17. **¹H-NMR spectrum of compound 18a.**

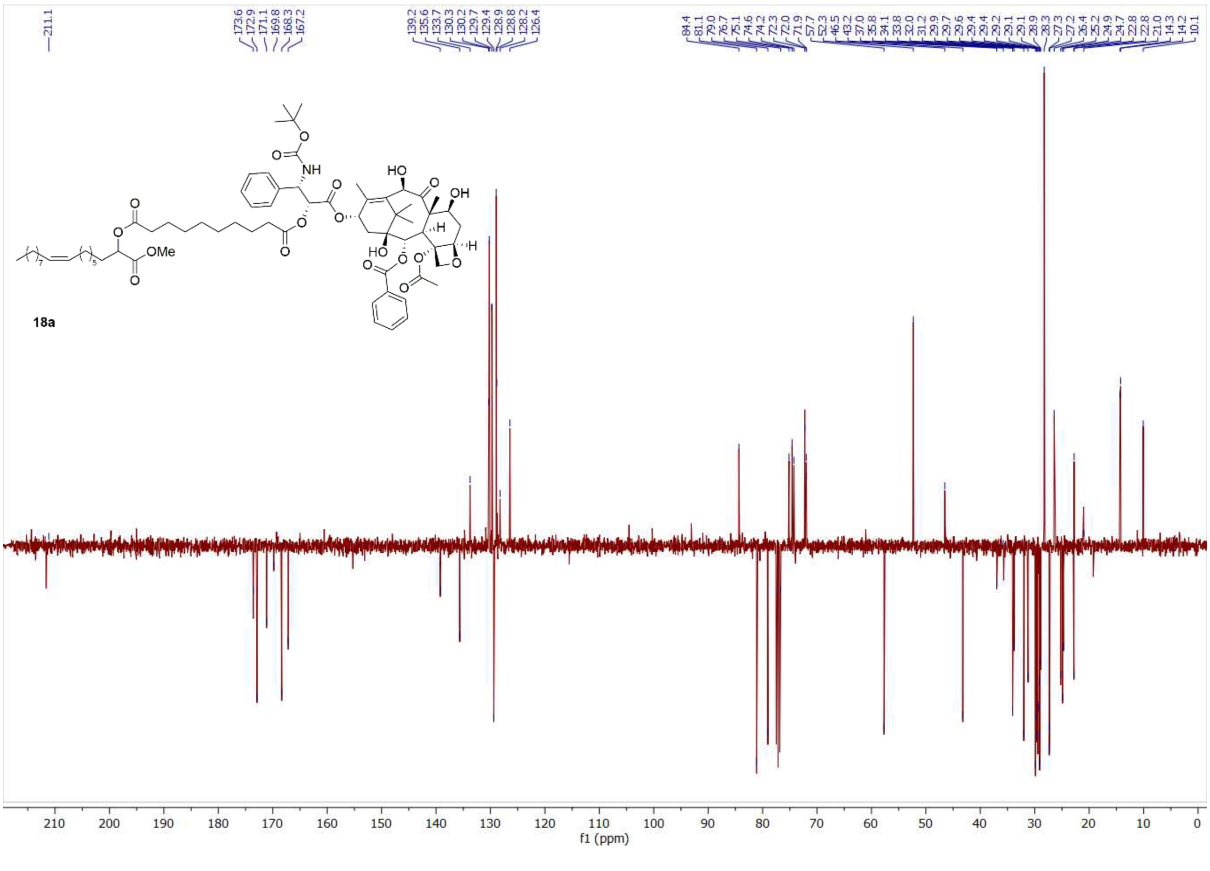

Figure S18. **¹³C-NMR spectrum of compound 18a.**

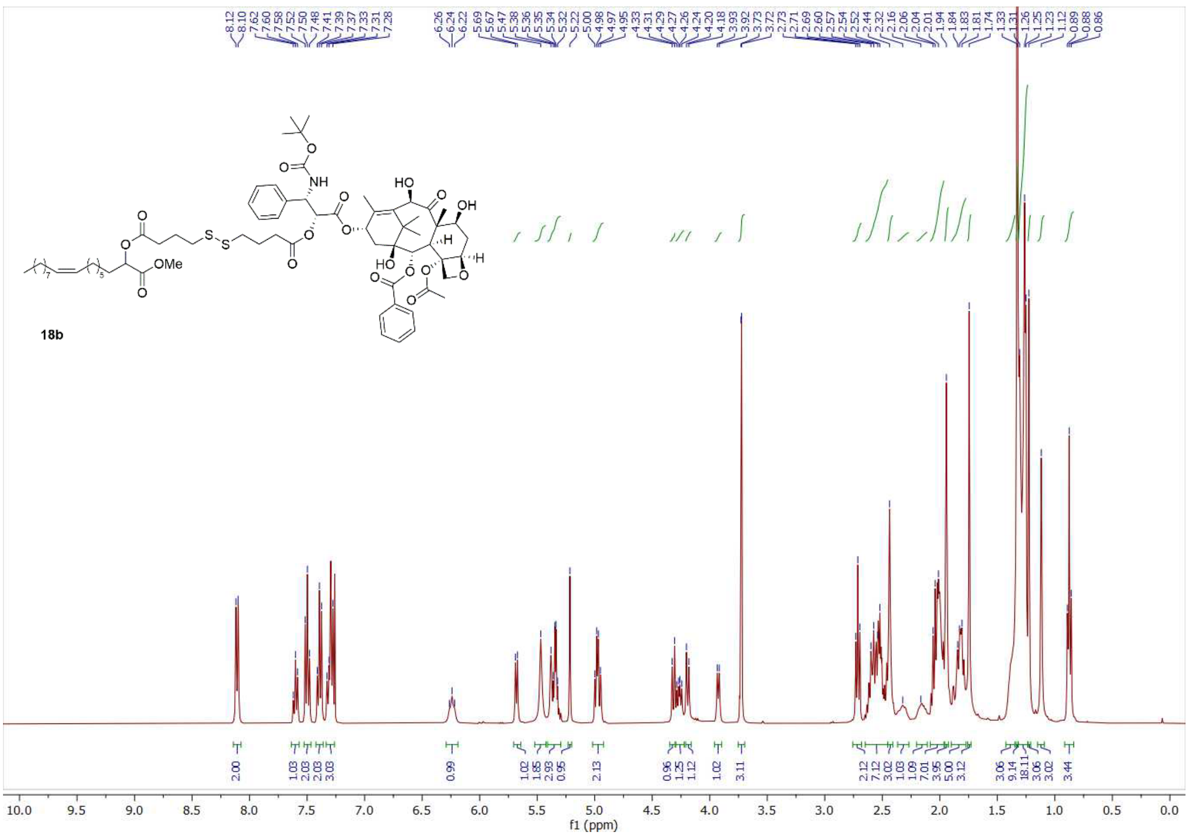

Figure S19. ^{1}H-NMR spectrum of compound 18b.

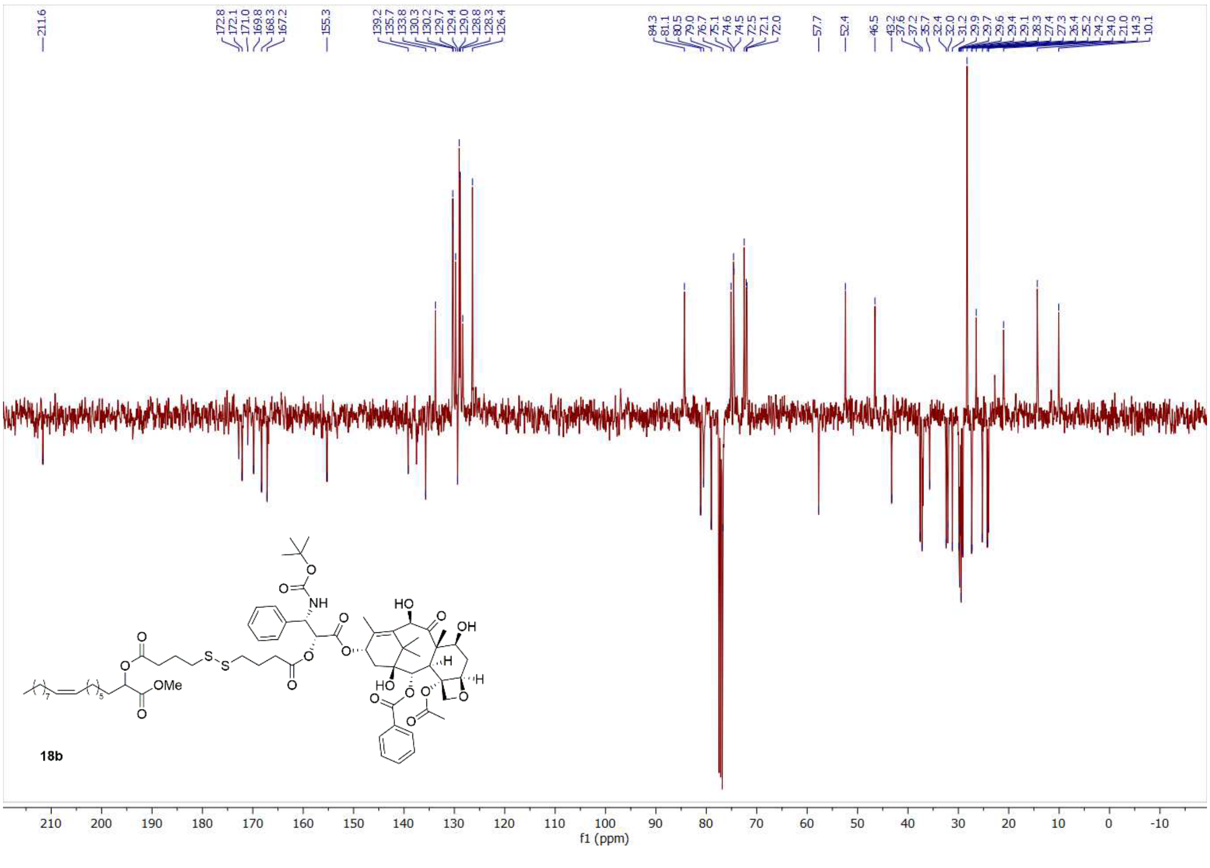

Figure S20. ^{13}C-NMR spectrum of compound 18b.

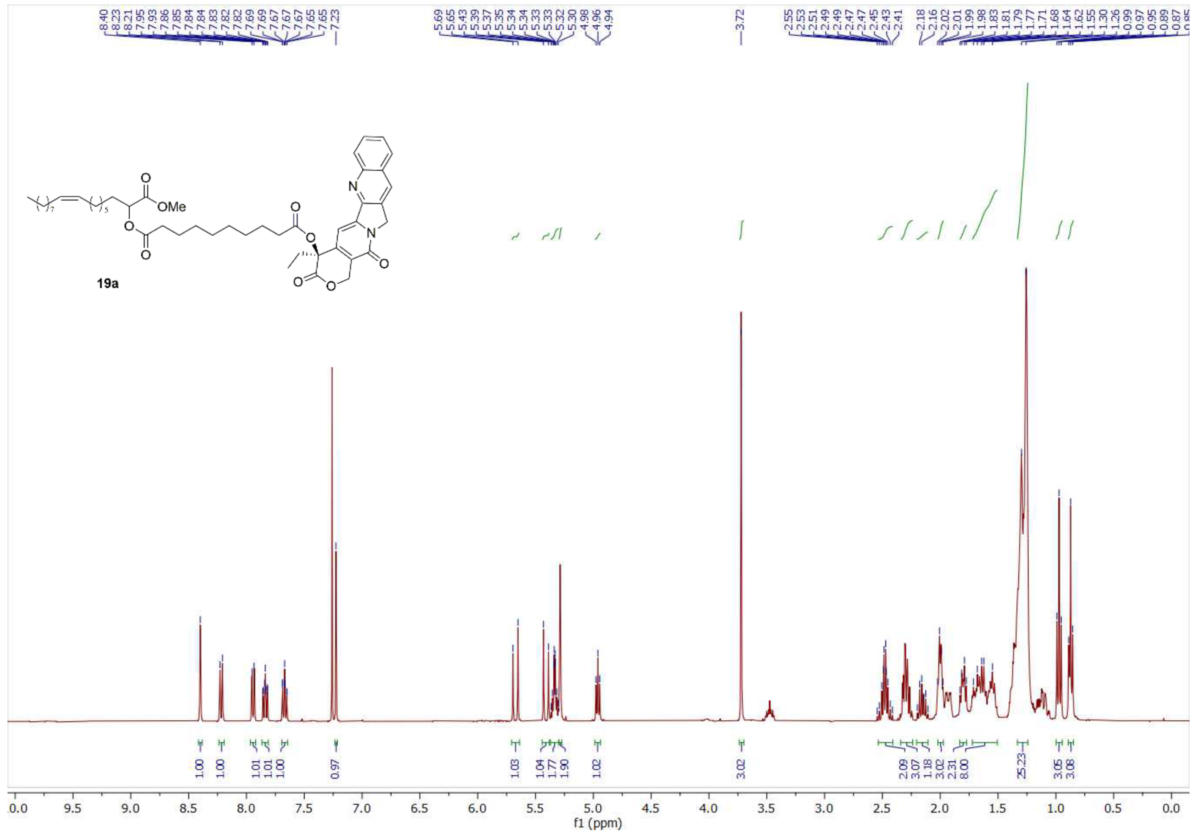

Figure S21. **^{1}H-NMR spectrum of compound 19a.**

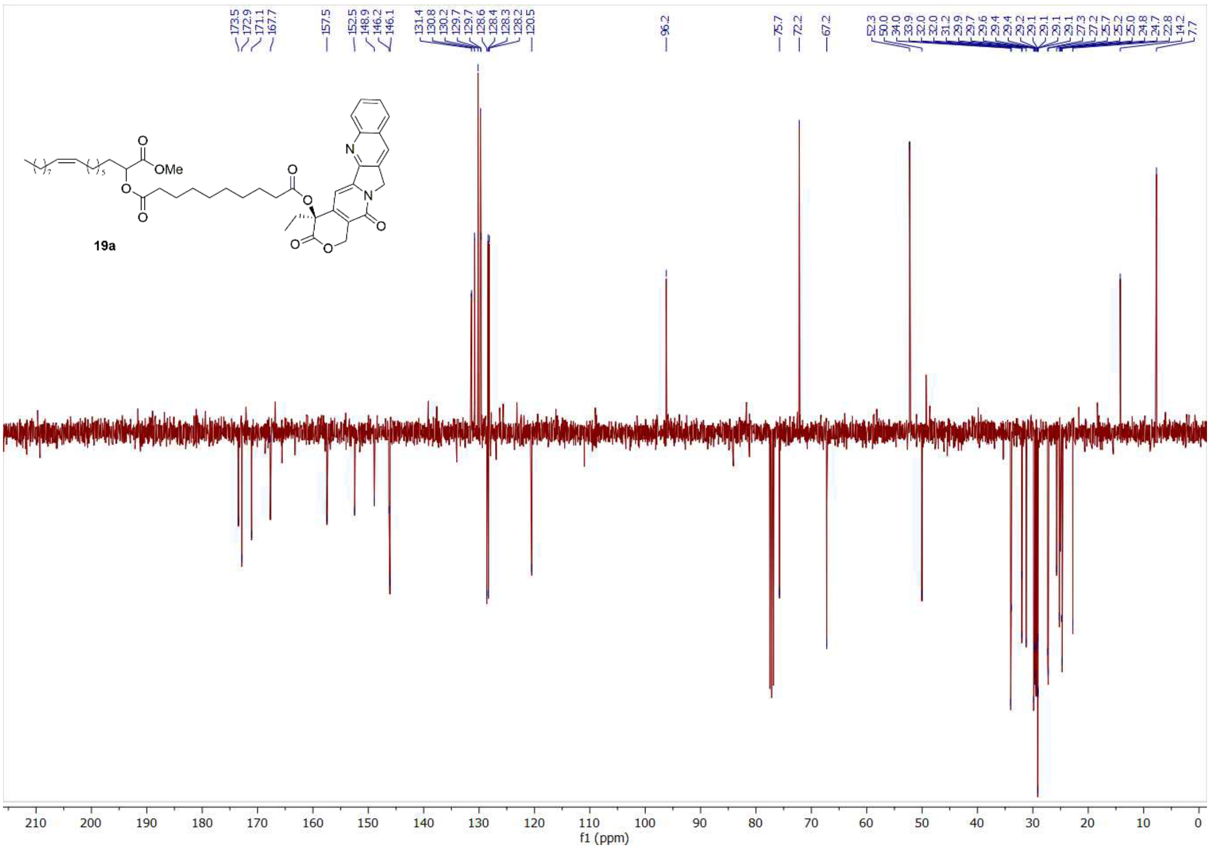

Figure S22. **^{13}C-NMR spectrum of compound 19a.**

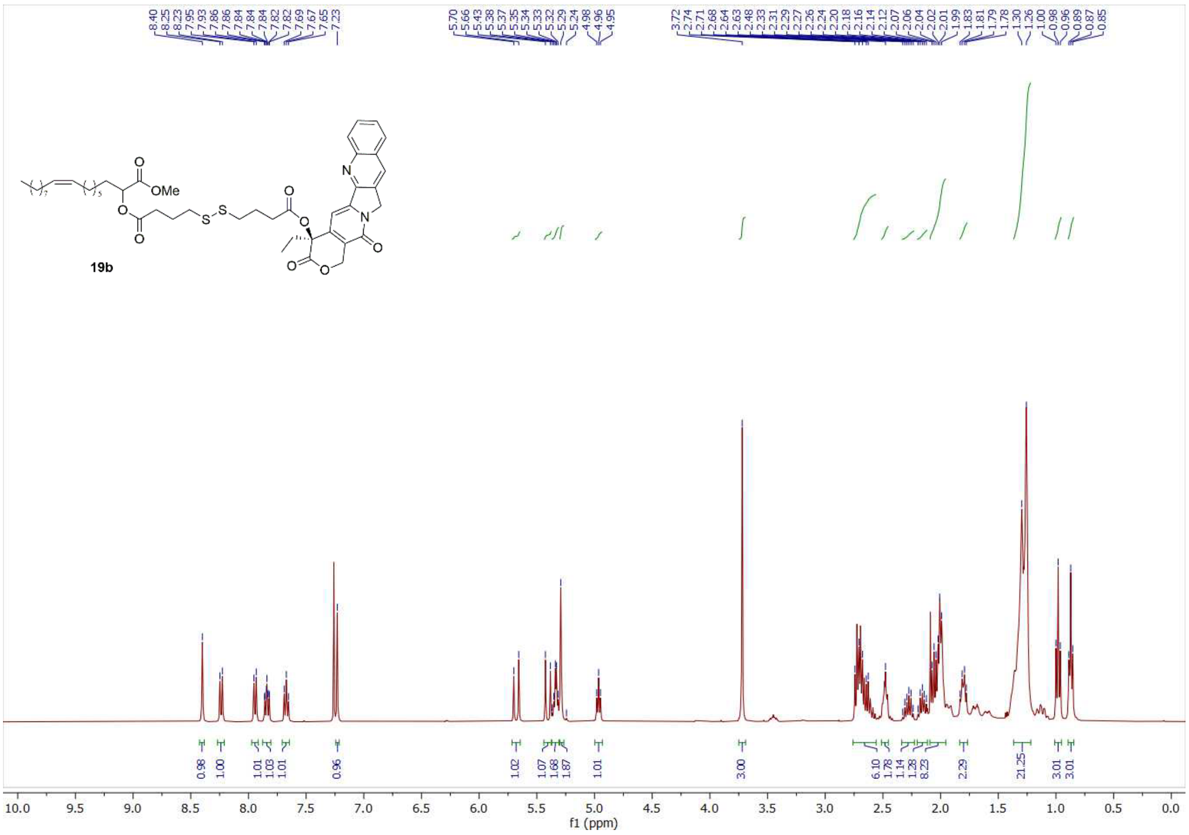

Figure S23. **^{1}H-NMR spectrum of compound 19b.**

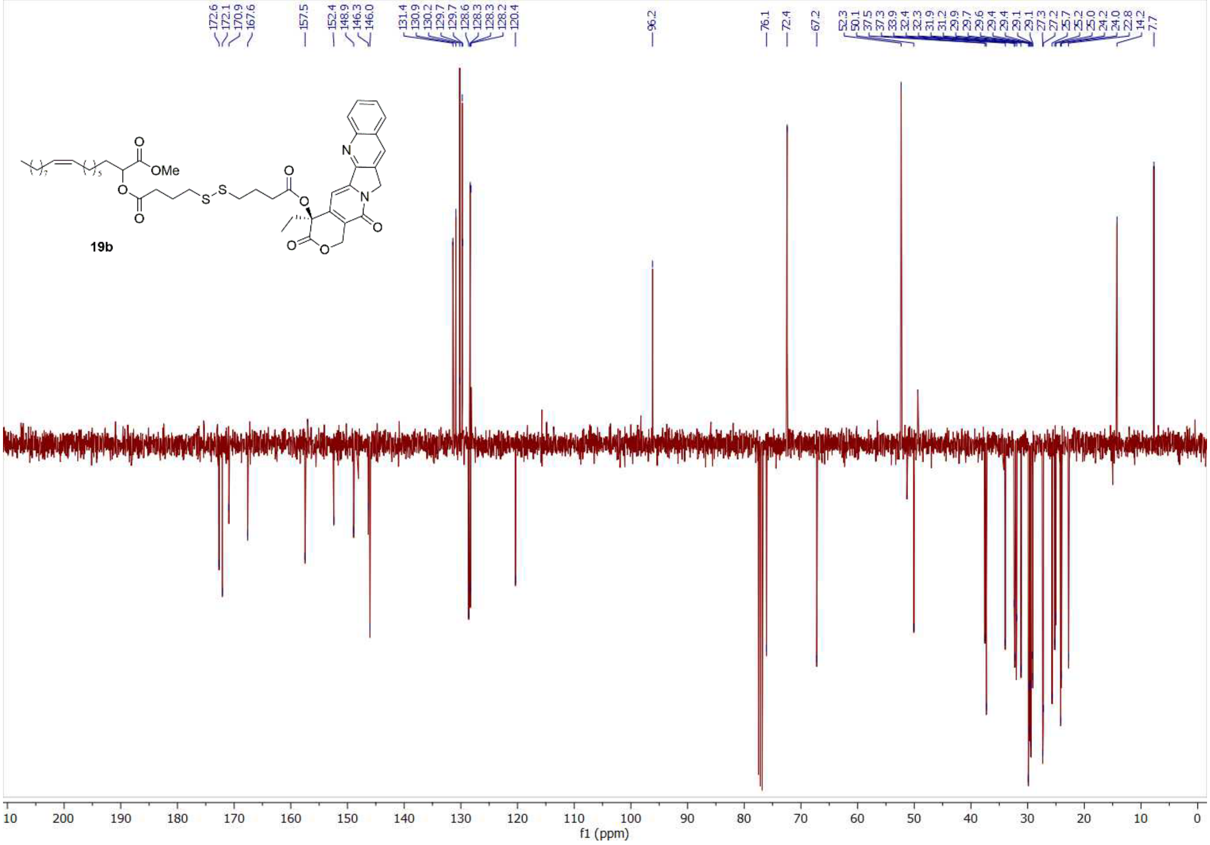

Figure S24. **^{13}C-NMR spectrum of compound 19b.**

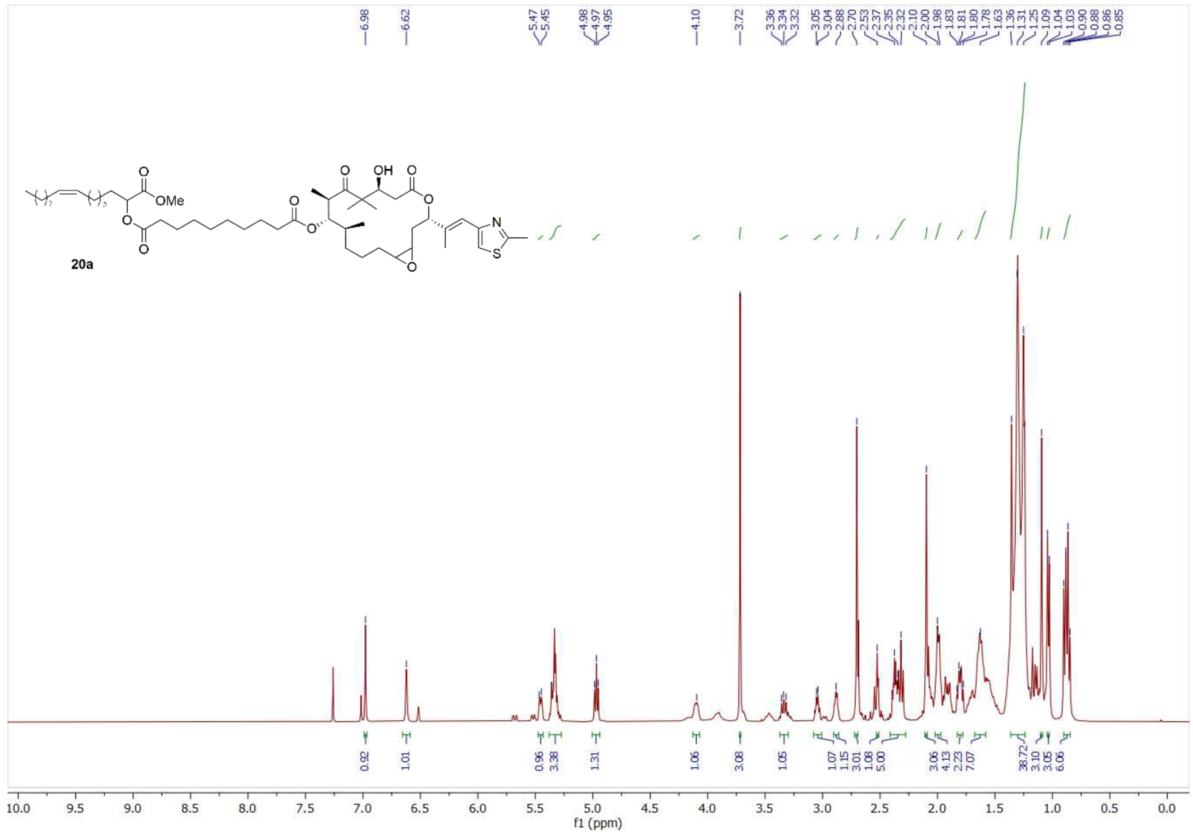

Figure S25. ¹H-NMR spectrum of compound 20a.

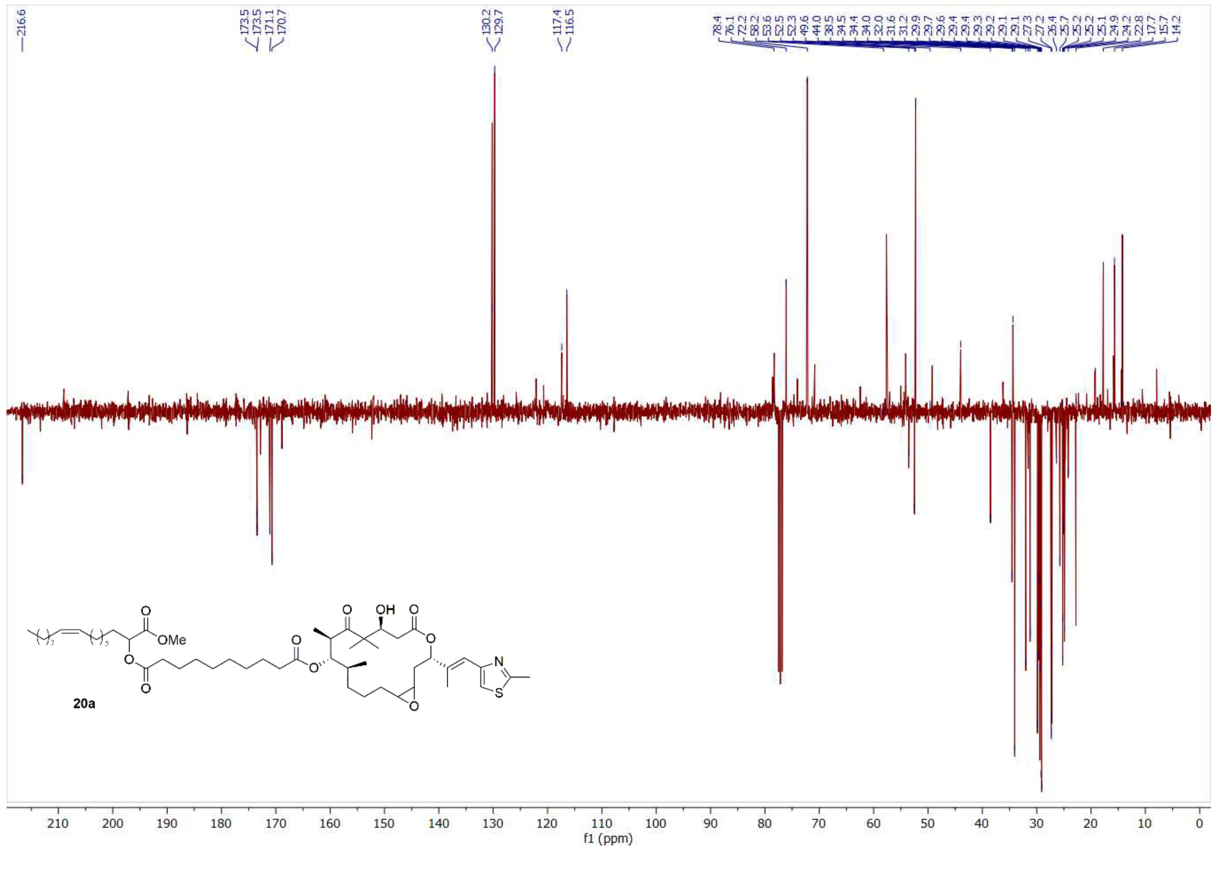

Figure S26. ¹³C-NMR spectrum of compound 20a.

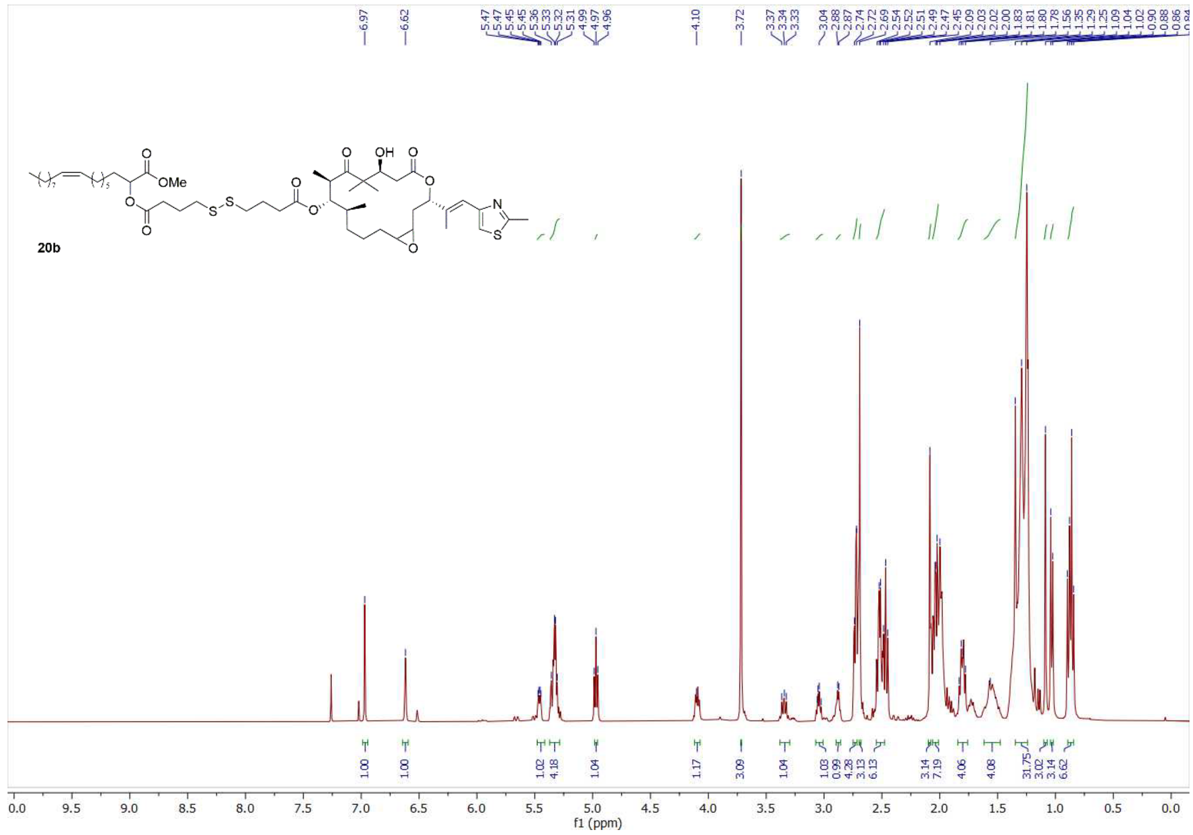

Figure S27. ^{1}H-NMR spectrum of compound 20b.

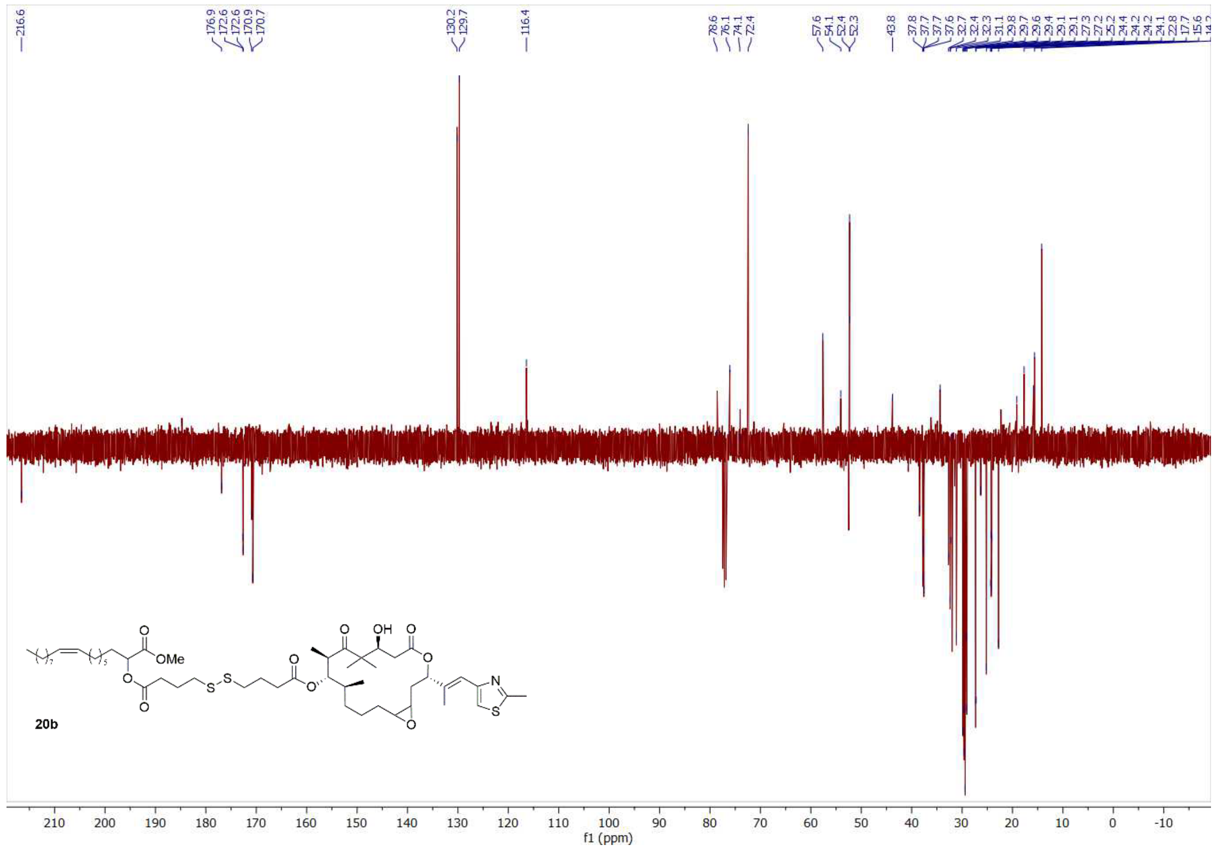

Figure S28. ^{13}C-NMR spectrum of compound 20b.

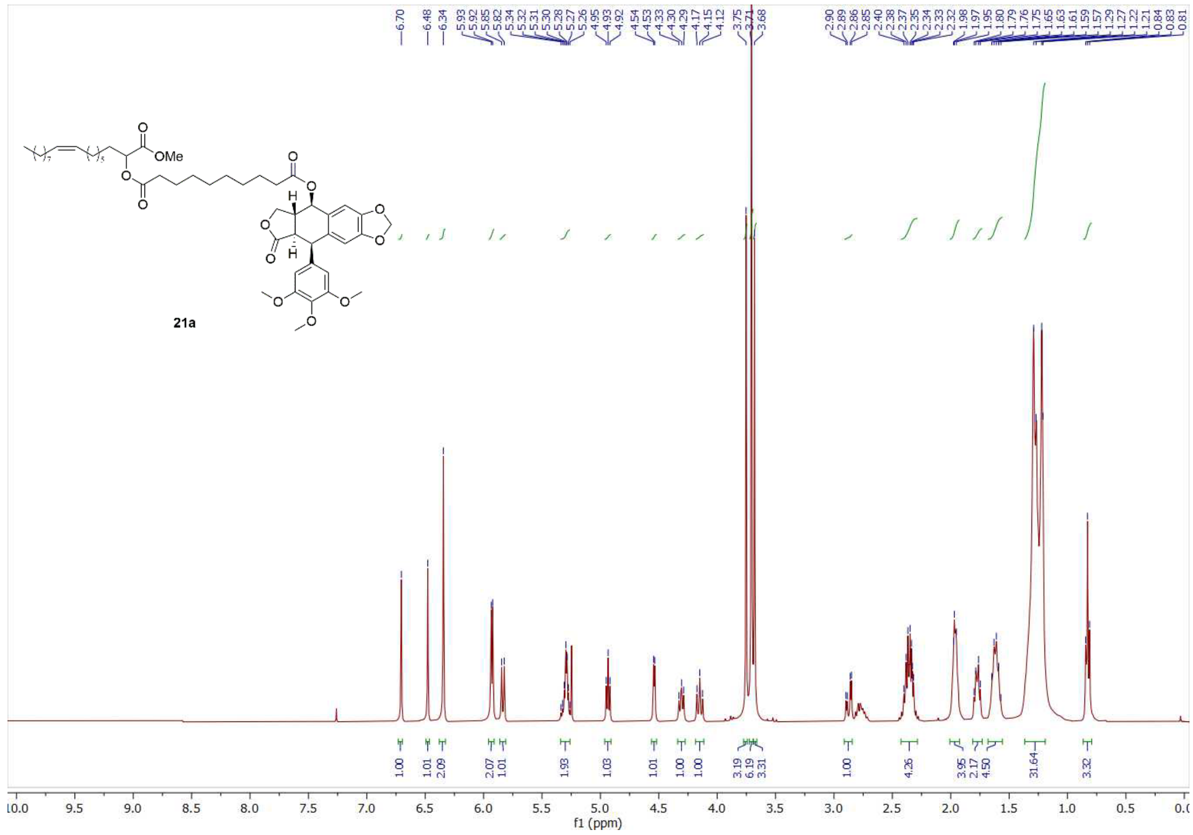

Figure S29. ¹H-NMR spectrum of compound 21a.

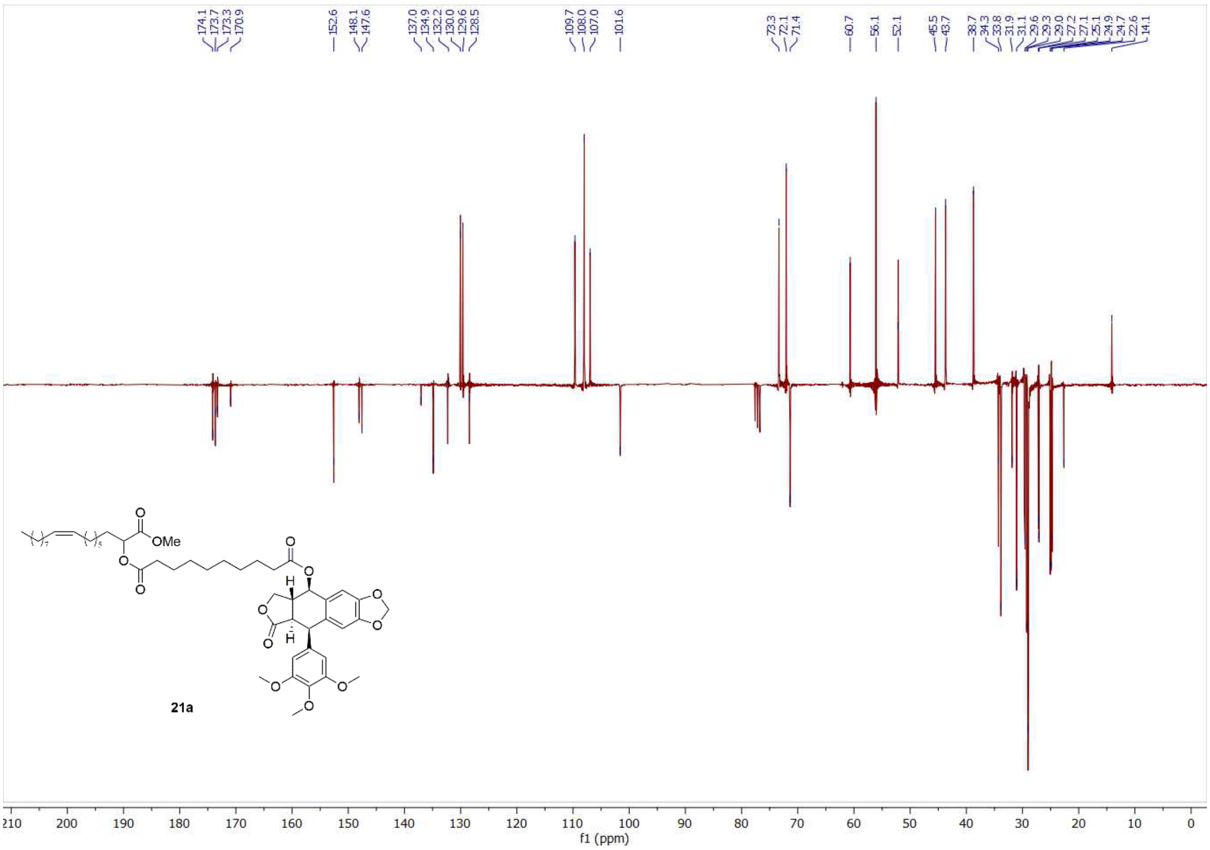

Figure S30. ¹³C-NMR spectrum of compound 21a.

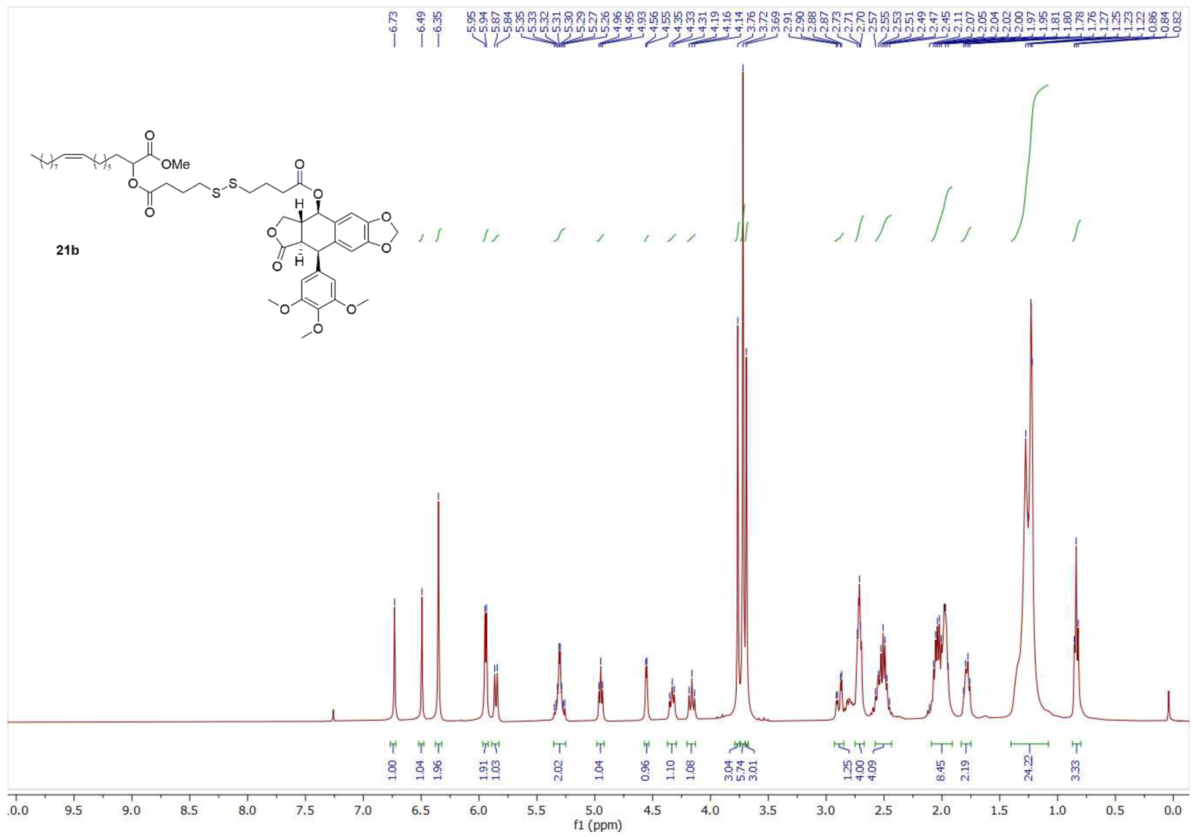

Figure S31. ^{1}**H-NMR spectrum of compound 21b.**

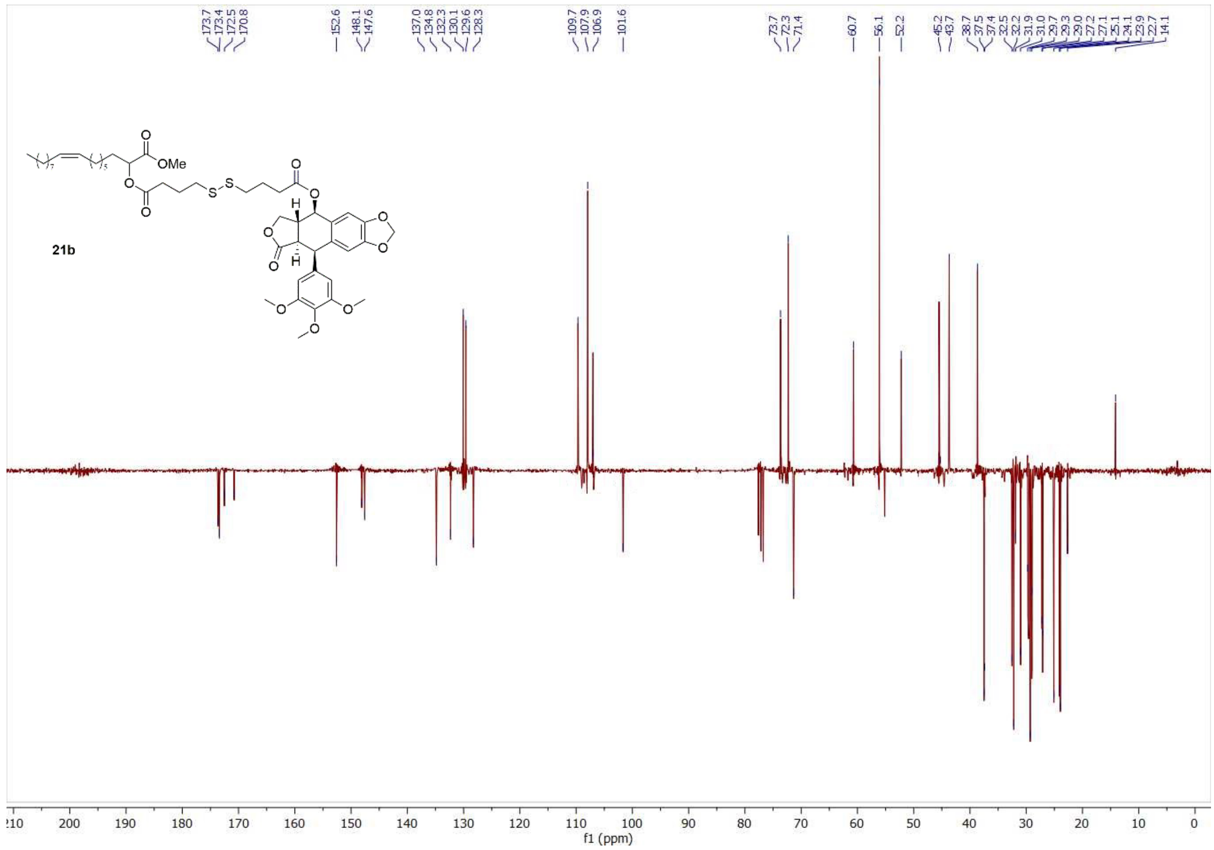

Figure S32. ^{13}**C-NMR spectrum of compound 21b.**

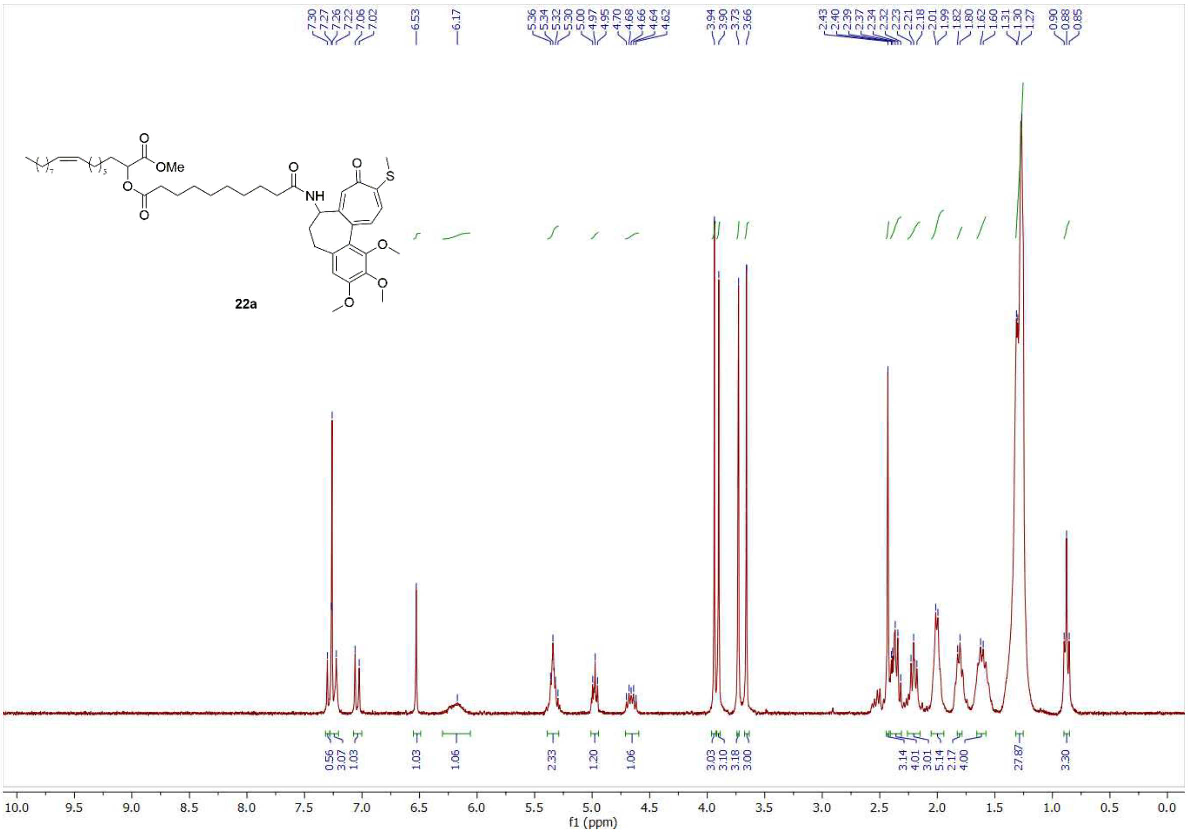

Figure S33. ^{1}H-NMR spectrum of compound 22a.

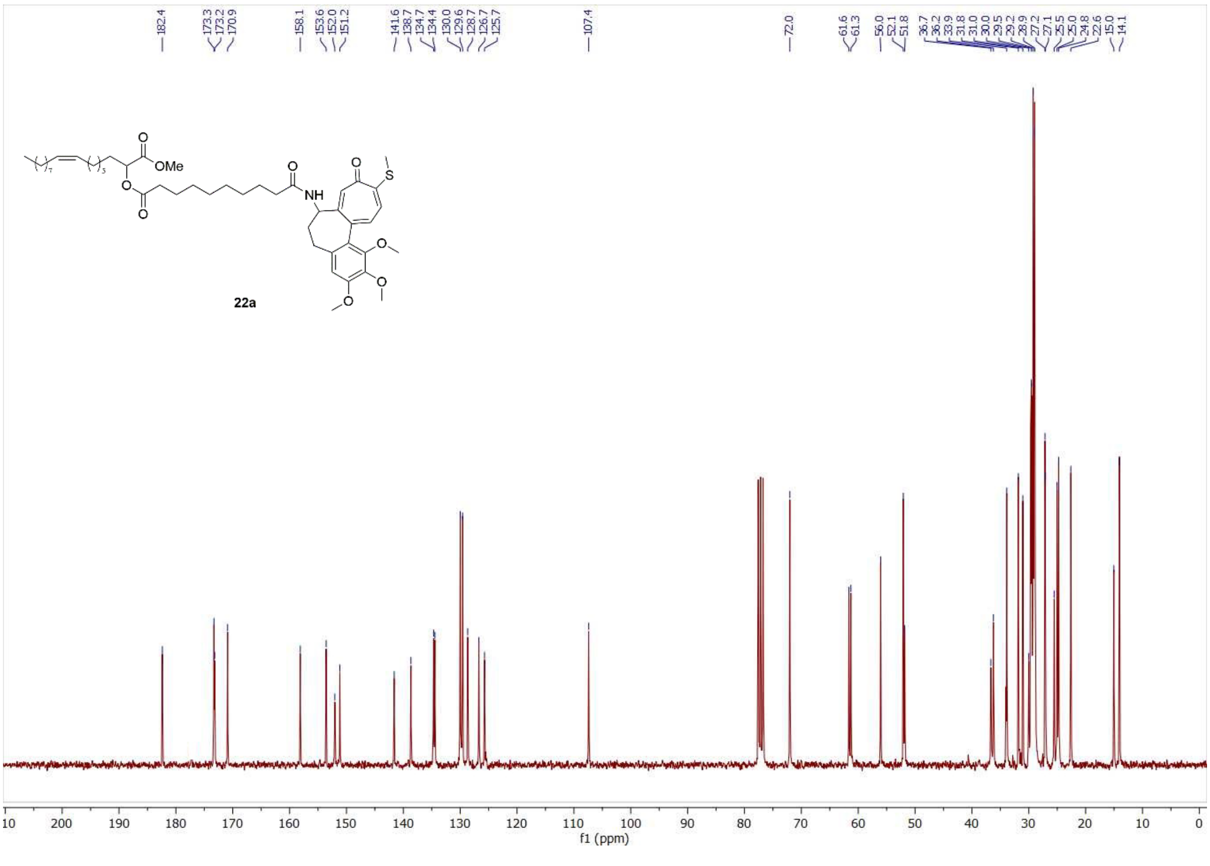

Figure S34. ^{13}C-NMR spectrum of compound 22a.

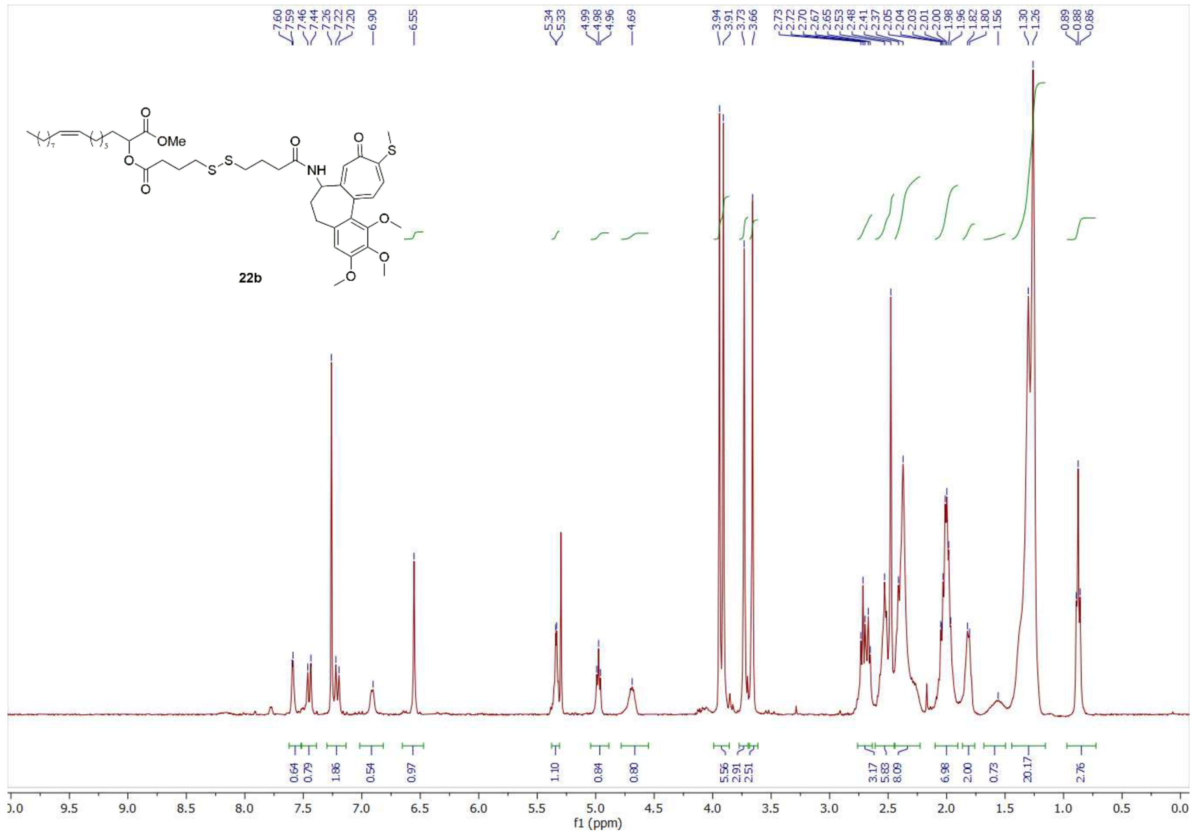

Figure S35. ^{1}H-NMR spectrum of compound 22b.

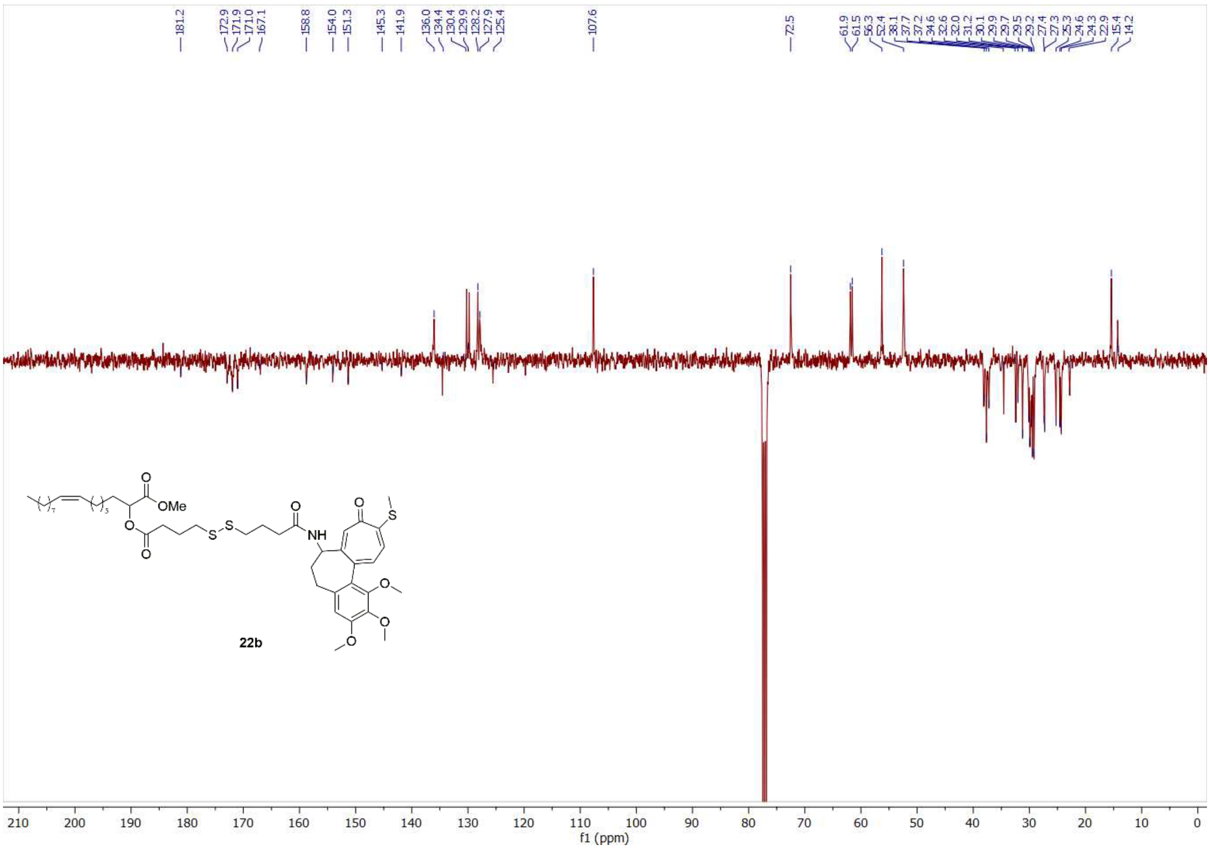

Figure S36. ^{13}C-NMR spectrum of compound 22

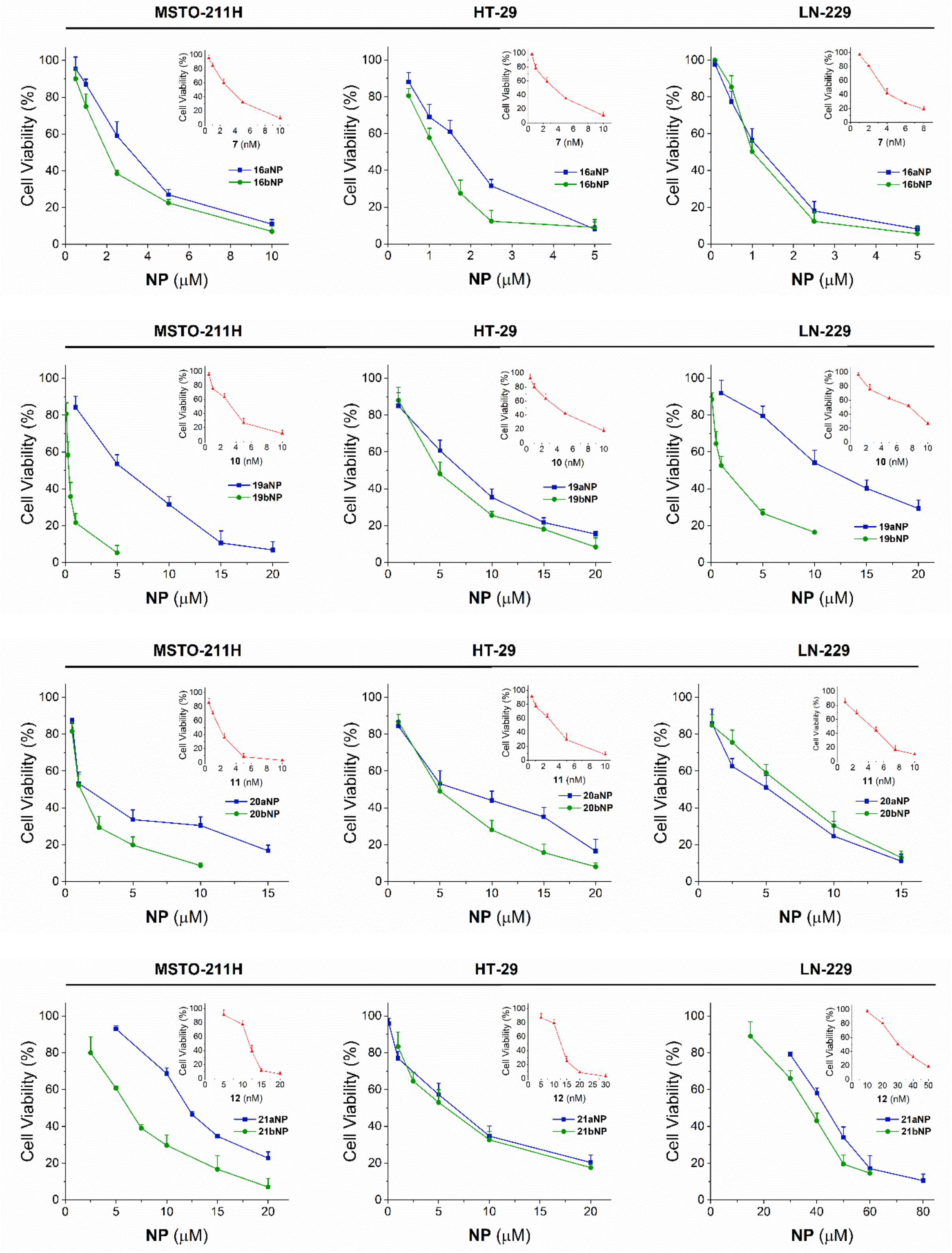

MSTO-211H
HT-29
LN-229
Cell Viability (%)
NP (μM)
7 (nM)
16aNP
16bNP
10 (nM)
19aNP
19bNP
11 (nM)
20aNP
20bNP
12 (nM)
21aNP
21bNP

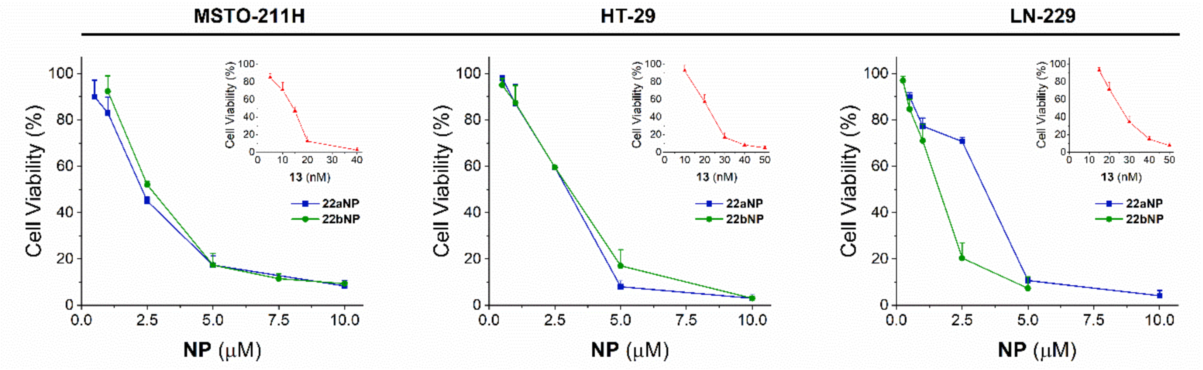

Figure S37. Cytotoxicity curves of compound 7, 10-13 and the corresponding nanoconjugates on MSTO-211H, HT-29 and LN229.
